# Humans 40,000 y ago developed a system of conventional signs

**DOI:** 10.1073/pnas.2520385123

**Published:** 2026-02-23

**Authors:** Christian Bentz, Ewa Dutkiewicz

**Affiliations:** ^a^Department of Language Science and Technology, Saarland University, Saarbrücken 66123, Germany; ^b^Faculty of Humanities and Cultural Studies, University of Passau, Passau 94032, Germany; ^c^Stone Age Department, Museum für Vor- und Frühgeschichte, Staatliche Museen zu Berlin, Berlin 10117, Germany

**Keywords:** Paleolithic signs, human evolution, quantitative linguistics, protocuneiform, information theory

## Abstract

As humans, we store and share information. This allows us to distribute knowledge necessary for survival and to coordinate large groups. Our hominin ancestors harnessed the surfaces of mobile artifacts and cave walls as information carriers since the Paleolithic time period. Theories abound as to the meaning and function of these Paleolithic signs. However, very little is known about their basic, measurable properties. We here analyze a corpus of more than 200 mobile objects of a 43,000 to 34,000 y old Aurignacian culture—associated with the first modern humans to settle in Central Europe. These objects are adorned with several thousand geometric signs. We apply classification algorithms and statistical models to capture their quantitative properties. First, our analyses illustrate that these sign sequences are clearly distinguishable from modern day writing. Second, however, their statistical properties are comparable to sign sequences on the earliest protocuneiform tablets. Third, Paleolithic signs were systematically applied to yield higher information density on certain types of objects, e.g. ivory figurines compared to tools. These results cannot be taken to strictly prove that Aurignacian sign sequences encoded numero-ideographic information as in the case of protocuneiform. However, they prove that the first hunter-gatherers arriving in Europe already applied sign sequences of comparable complexity in a deliberate, systematic, and conventional manner—several ten thousand years before the advent of genuine writing.

## Significance

Humans have carved visual signs into the surfaces of mobile artifacts and cave walls since several hundred thousand years. We here analyze a 40,000 y old assemblage of mobile artifacts bearing sequences of intentionally engraved geometric signs. These sign sequences have a complexity comparable to the earliest protocuneiform and were selectively applied to yield higher information density on figurines than on tools. This proves that the first hunter-gatherers arriving in Europe already developed a system of intentional and conventional signs on mobile artifacts. Our study more broadly relates to research into statistical properties of human language and writing compared to other sign systems.

As humans, we store information outside of our minds. This capacity for information encoding beyond the here and now of spoken words is nowadays reflected in symbols and written language. It is a hallmark of human cognitive evolution and underlies artificial computing systems. Without it, the information age would not be possible. Archaeologists have identified reflections of this capacity in excavation layers dating back to the Paleolithic period of 400,000 to 15,000 thousand years ago (1–5).

At the beginning of the Upper Paleolithic around 45,000 calibrated years before present (cal BP), modern humans arrived in Eastern and Central Europe (6, 7). On their journey they encountered their distant relatives—Neanderthals (8–11). In this time of migrations and population turn-overs, modern humans produced a panoply of so-called mobile objects, such as tools and figurines made of ivory, bone, or antler. These are present right from the earliest period of the Upper Paleolithic, the so-called Aurignacian technocomplex. Especially the Dordogne region in southwestern France (12–14), the cave systems of the Swabian Jura in southwestern Germany (15–17), and a cluster of sites in Belgium (18, 19) have yielded hundreds of objects adorned with sequences of geometric signs.

In earlier studies, selected artifacts were analyzed microscopically and experimentally to demonstrate that so-called artificial memory systems (AMS) might have their beginnings already in the late Middle Paleolithic associated with Neanderthals of 70,000 to 40,000 y ago (3, 20, 21). Namely, a few mobile artifacts (e.g. raven and hyena bones) bear regular incisions potentially encoding numerical information. Some traces of artificial memory systems or “exograms” might even reach back to the Lower Paleolithic several hundred thousand years ago (22, 23).

Having said this, there is a clear increase in the number of mobile artifacts with geometric markings in assemblages of the Upper Paleolithic period (5, 12–14, 16, 24). Moreover, the codes of artificial memory systems seem to have become more complex toward the end of this period (3). How to exactly quantify and model this complexification remains unclear. We here provide openly published data and computational tools to assess the complexification of sign sequences produced by humans over evolutionary time.

In general, our study builds on research applying computational tools to pin down the statistical properties of human language in comparison to ancient sign systems (25–32) as well as animal communication systems (33–36). More specifically, it relates to archaeological investigations of Paleolithic signs on mobile objects (3, 17, 20, 21, 32, 37) as well as cave art (38–42)—sometimes explicitly linked to numerical cognition (43). Also, experimental work emerges to understand how humans perceive the geometric patterns on mobile artifacts and parietal art, and to clarify their semiotic status (44–47). This is embedded more generally into experimental work on the evolution of semiotic systems in the lab (48–50), as well as the cognitive biases shaping them (51, 52). Our study can thus be seen as part of an emerging field of research: Evolutionary Semiotics.

## Signs of the Swabian Aurignacian.

Our analyses are based on assessment of overall 260 mobile artifacts from the Swabian Aurignacian (17)—a cluster of cave sites in southwestern Germany. The people inhabiting these caves between 43,000 to 34,000 y cal BP (53) have produced a specialized range of tools to cut meat, work animal hides, and create clothes and ropes (54). They have developed the first musical instruments—flutes—made of bones and ivory (55). Moreover, they have left behind symbolic artifacts, such as beads and pendants for personal ornamentation which bear a local signature (56). Finally, the assemblages are enriched by several dozen figurines laboriously carved out of ivory, reflecting the natural environment as well as a spiritual world unknown to us. This includes animal species living in the area at the time such as woolly mammoths (*Mammuthus primigenius*), wild horses (*Equus ferus*), steppe bison (*Bison priscus*), cave bears (*Ursus spelaeus*), cave lions (*Panthera spelaea*), etc. Besides animal depictions, we also find anthropomorphic depictions including female figurines (57), as well as hybrid creatures between human and cave lion (58).

We are interested in the sign sequences carried by these mobile artifacts. As the most general definition of a “sign” in the context of archaeological material we propose: a modification of a surface which can be perceived and interpreted by an intelligent viewer. Note that this includes bite marks, cut marks, utilitarian manipulations, etc. In our analyses, we restrict this further to intentional and nonutilitarian signs. “Intentional” means that they are not the by-product of another activity, e.g. cut marks as a result of butchering. “Nonutilitarian” means there is no indication that these surface modifications had purely practical functions relating to craftsmanship, e.g. holes for attaching ropes, or circular incisions to help with hafting of spear points. The inventory of sign types includes basic shapes such as lines, points, crosses, but also more complex patterns such as stars, grid patterns, and zigzag lines.

**Fig. 1.**
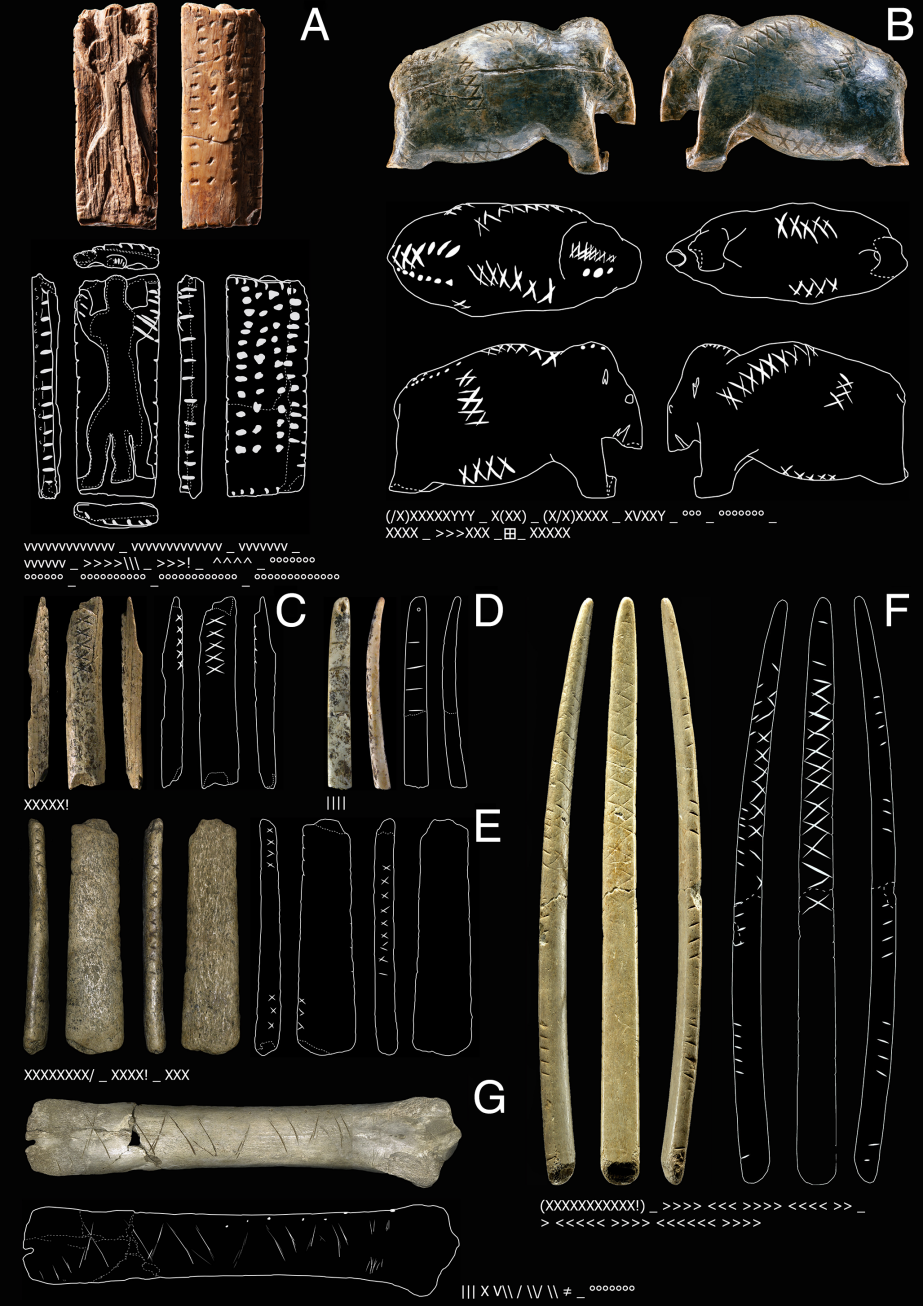
Mobile artifacts with geometric signs of the Swabian Aurignacian. (*A*) Plaquette with hybrid creature (so-called “Adorant”), ivory, Geissenklösterle (gkl0025), © Landesmuseum Württemberg, Hendrik Zweitasch. (*B*) Mammoth figurine, ivory, Vogelherd (vhc0145), © University of Tübingen, Juraj Lipták. (*C*) Rod/bâton, ivory, Vogelherd (vhc0001), © University of Tübingen, Ewa Dutkiewicz. (*D*) Personal ornament, ivory, Geissenklösterle (gkl0006), © University of Tübingen, Ewa Dutkiewicz. (*E*) Spatula/lissoir, bone, Vogelherd (vhc0017), © University of Tübingen, Ewa Dutkiewicz. (*F*) Spatula/lissoir, bone, Vogelherd (vhc0162), © University of Tübingen, Juraj Lipták. (*G*) Undetermined, bone, Hohle Fels (hfc0006), © University of Tübingen, Ewa Dutkiewicz. Drawings by Ewa Dutkiewicz. Copyright: CC-BY-SA 4.0. For further details on sign coding and preprocessing see *Materials and Methods*.

The assignment of sign types to UTF-8 characters is based on first-hand microscopic analyses of the second author. Further details on the materials and analyses for the artifacts of the Swabian Aurignacian are published (17). The publication of the first version of the database (5) includes an evaluation of sign type codings given alternative choices by other researchers who are familiar with the material. The agreement scores for alternative sign type assignments are 91 to 94%, and Cohen’s Kappa scores are 0.29 to 0.44. Further details and examples are given in *Materials and Methods*. Sequences of signs are found on the surfaces of different types of mobile artifacts (Fig. 1), and they are particularly pervasive in four cave sites of the Swabian Aurignacian which are within hiking distance of one another (*SI Appendix*, Fig. S1).

**Fig. 2.**
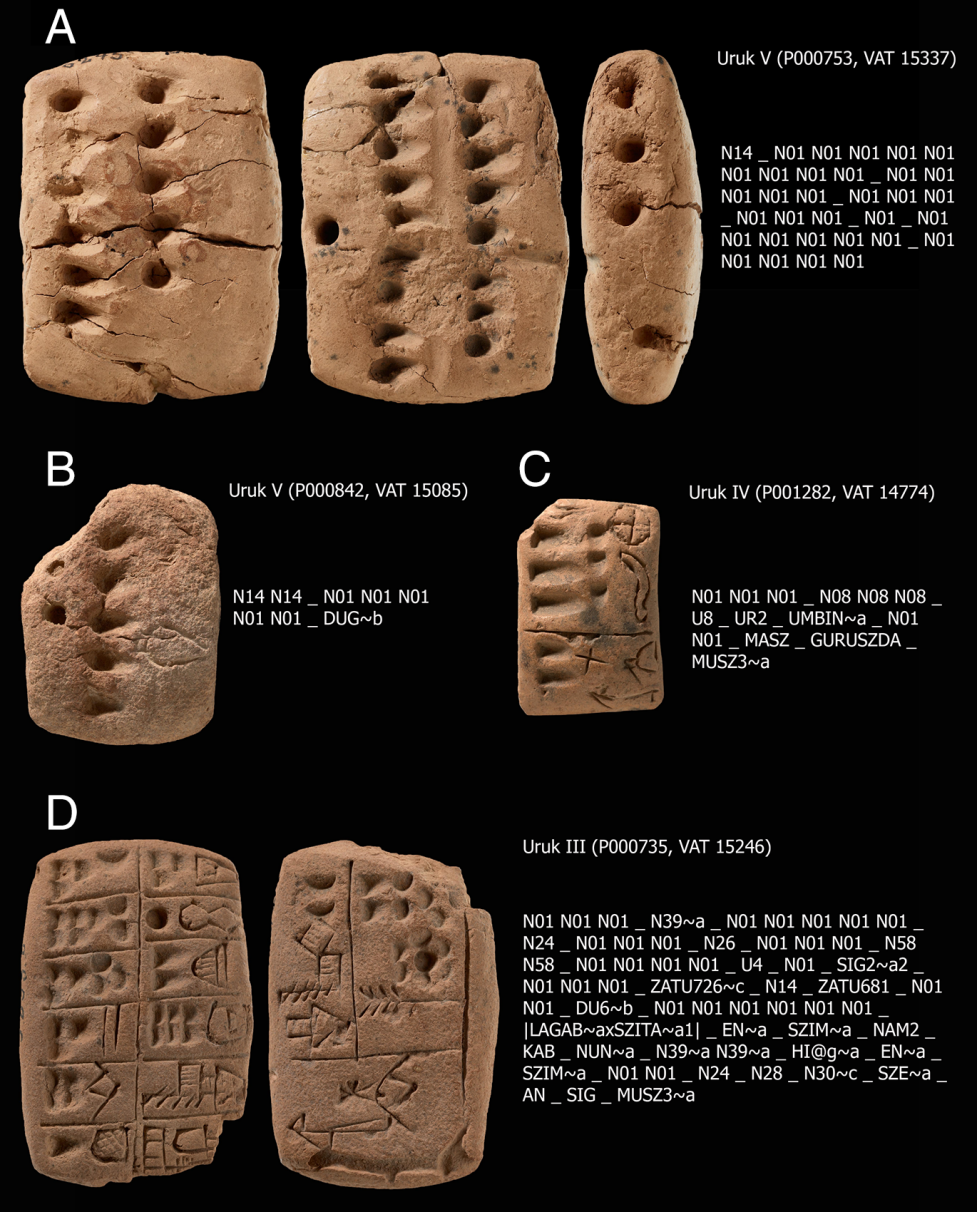
Examples of Uruk protocuneiform tablets from Uruk V to Uruk III. The CDLI identifiers and collection numbers are given in parentheses. The preprocessed version of the transliteration of protocuneiform signs according to CDLI is given to the right of a tablet. The transliterations for different faces of the tablet (reverse, obverse) are here simply concatenated. (*A*) Uruk V tablet (CDLI identifier: P000753). (*B*) Uruk V tablet (CDLI identifier: P000842). (*C*) Uruk IV tablet (CDLI identifier: P001282). (*D*) Uruk III tablet (CDLI identifier: P000735). Copyright: CC-BY-SA 4.0, *Staatliche Museen zu Berlin, Vorderasiatisches Museum/Olaf M. Teßmer*.

As comparative samples we employ material from the earliest periods of protocuneiform in ancient Mesopotamia: Uruk V (approx. 3500–3350 BC), Uruk IV (approx. 3350–3200 BC), and Uruk III (approx. 3200–3000 BC). See Fig. 2 for example tablets. We also harness the Text Data Diversity (TeDDi) sample of 89 different languages written in 16 scripts (59) as a modern day counterpart.

Based on these samples we, first, estimate statistical features from quantitative linguistics for all the approx. 3000 sign sequences in these corpora: type-token-ratios, unigram entropies, entropy rates, and repetition rates. See *SI Appendix*, Fig. S2 for an overview of the workflow. Second, we input the feature values to classification algorithms. This enables us to assess how similar or different the sequences are given their labels, i.e. Aurignacian, protocuneiform, and modern day writing. Third, we fit multiple regression models for the Aurignacian sequences to predict their information density given meta-information, i.e. the type of artifact (tool, figurine, personal ornament, and others), the volume of an artifact, its preservation, its age, etc. For further details see *Materials and Methods*.

## Results

### Statistical Feature Distributions.

**Fig. 3.**
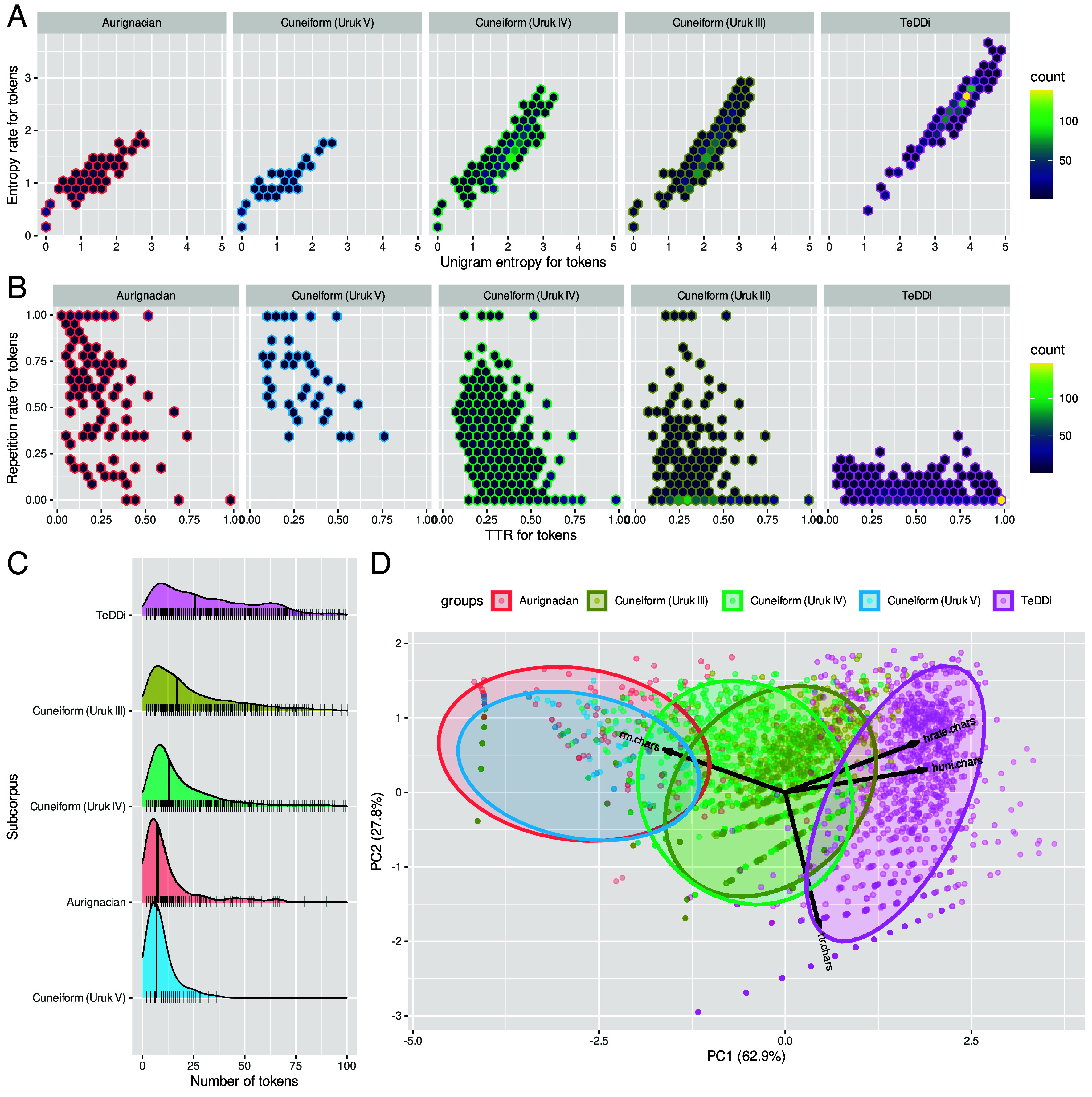
Statistical feature distributions. (*A*) Hexagonal bin plots of unigram entropies and entropy rates for Aurignacian, protocuneiform, and modern writing character sequences. The x-axis and y-axis are divided into 20 bins. The color of each bin reflects the data point count in it. (*B*) Type-token-ratios (TTR) and repetition rates. (*C*) Distributions of sequence lengths per subcorpus. Black vertical lines give median values. Each vertical tick on the x-axis represents the length of a sequence in UTF-8 characters. (*D*) Scatterplot of data points for the first two principal components of a PCA on the four statistical features. Normal data ellipses are given colored by the respective group. Arrows represent the correlations of the statistical feature variables with PC1 and PC2.

Fig. 3 *A* and *B* gives the distributions of estimated feature values for the different subcorpora. Aurignacian sequences as well as Uruk V sequences have low unigram entropies and entropy rates paired with relatively high repetition rates. For the later Uruk IV and III periods, entropies successively incline—mirrored by a decline in repetition rates. Modern day writing systems are then characterized by high entropies and low repetition rates. In terms of length in number of tokens (Fig. 3*C*), Aurignacian and Uruk V sequences are again very similar (median values: $\tilde{\mu }=8$ and $\tilde{\mu }=7$), while lengths increase for the Uruk IV and Uruk III periods ($\tilde{\mu }=13$ and $\tilde{\mu }=17$), and achieve the highest values for modern day writing ($\tilde{\mu }=28$). Note, however, that the correlations between the sequence lengths and the four statistical features are small to medium (see correlogram in *SI Appendix*, Fig. S3). Hence, sequence lengths by themselves are only mediocre predictors of the statistical features.

When the four dimensions of statistical features are projected into two dimensions via PCA (Fig. 3*D*), the normal data ellipses of the Aurignacian data and the Uruk V data fully overlap. Both also marginally overlap with Uruk IV and Uruk III ellipses—but not with the modern writing (TeDDi) ellipsis. Uruk IV and III are in the middle ground between Aurignacian/Uruk V, on one hand, and modern writing, on the other.

### Classification of Sign Sequences.

The results of classification analyses are provided in *SI Appendix*, Fig. S4. Both Multi-Layer Perceptrons (MLP) and K-Nearest-Neighbors (KNN) perform similarly. Namely, the accuracies achieved for distinguishing between Aurignacian and Uruk V sequences are generally not significantly different from the “no information rate” baseline. In other words, the classifiers do not perform better than an algorithm which would just assign the most frequent label in the training set to all sequences of the test set. When distinguishing between Aurignacian and Uruk IV/Uruk III sequences, accuracies increase and are often higher than the baseline. Finally, for distinguishing between Aurignacian and modern writing the algorithms generally achieve maximal accuracy (close to 100% on average), being significantly different in general from the baseline.

### Multiple Regression Models Predicting Information Density.

**Fig. 4.**
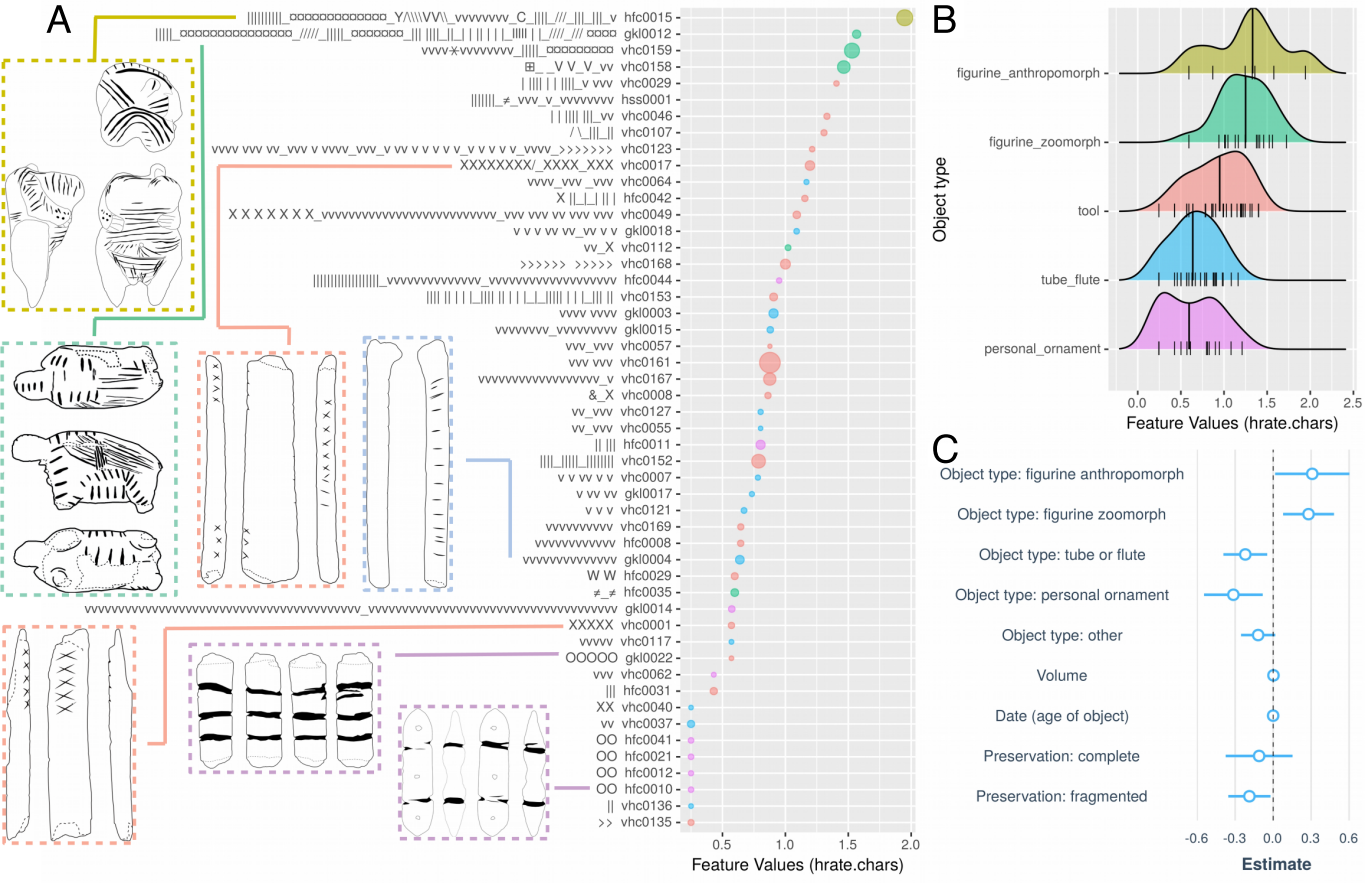
Results of multiple regression analyses. (*A*) Sequence plot. Aurignacian sign sequences (50 out of the overall 213 randomly sampled for visual illustration) with their respective entropy rates (x-axis). Colors indicate the type of object carrying a given sequence. The size of dots reflects the volume of the object (in cm^3^). (*B*) Entropy rate distributions per object type. Black vertical lines represent median values. (*C*) Coefficient plot. Estimated coefficients for a multiple linear regression model predicting entropy rate per sequence of a given object (response variable) from object type (factor variable, reference level “tool”), material (factor variable, reference level “antler”), estimated volume of object (continuous variable), and preservation of object (factor variable, reference level “almost complete”). The dots represent coefficient values, the blue lines are 0.95 CIs. A given coefficient is significantly different from zero if the CI does not intersect with the dashed line.

The results of multiple regression models predicting entropy rates from meta-information about the objects are visualized in Fig. 4. In the best model in terms of the Akaike Information Criterion (AIC), the significant coefficient estimates mainly derive from the object type variable (F=8.5, df=196, P<0.001, $R^{2}=0.25$). Namely, the so-called semipartial r-squared value ($R^{2}$ value difference between a model with and without object type as predictor) is significantly higher than zero: $\Delta R^{2}=0.13,CI=\left[0.07,1\right]$. More precisely, the estimates for anthropomorph figurines are positive (β=0.29, t=1.95, P=0.05) and borderline significant, and those for zoomorph figurines are positive (β=0.23, t=2.4, P=0.02) and significant. This means they are significantly higher compared to the reference level of tools. The estimates for tubes/flutes (β=−0.22, t=−2.5, P=0.012) and personal ornaments (β=−0.32, t=−2.8, P<0.01), on the other hand, are significantly lower than for tools. Since entropy rates for Aurignacian sequences in general are roughly in the range [0,2], these coefficient estimates mean that ivory figurines tend to carry sequences of around 15% higher information density than tools. Tools, in turn, have around 10% higher information density than tubes/flutes, and around 15% higher than personal ornaments.

These effects cannot be driven by differences in the volumes or the state of preservation of objects, since these are included in the model as alternative predictors. The volume of objects is associated with a positive coefficient, i.e. bigger objects tend to have higher information density. However, this effect is small, borderline significant, with a nonsignificant effect size (β=0.003, t=1.7, P=0.08, $\Delta R^{2}=0.01$). Regarding preservation, we find that fragmented objects tend to have around 10% lower information density (β=−0.2, t=−2.4, P=0.02, $\Delta R^{2}=0.02$) than objects with the reference level “almost complete.”

All other predictors (and interactions between these) which were considered in different versions of the model are not significant and have nonsignificant effect sizes. This also includes the factorial predictor of cave sites with reference level “Geissenklösterle” against the other levels with sufficient data points (“Hohle Fels,” “Vogelherd”). Interestingly, the effect of the estimated maximum date before present (BP) of an object (ranging roughly from 40,000 to 30,000) is also not a significant predictor of entropy rate (β=−0.00002, t=−1.3, P=0.19, $\Delta R^{2}=0.006$). In other words, the sign sequences are relatively stable in terms of their information density over roughly 10,000 y. Model specifications and further statistics are given in *Materials and Methods*.

## Discussion

Against the backdrop of our results, we discuss common questions raised about mobile artifacts with markings of the Swabian Aurignacian in particular, and the Upper Paleolithic period more generally.

### The Question of Decoration.

Recent studies have measured the regularity of notches on bones to determine whether they are more or less visually striking as a decoration (3, 20). Increasing the regularity of distances between notches—up to the differences just about perceivable by humans—is argued to enhance the decorative value. Such technological and experimental analyses are useful to thoroughly understand the production processes behind a given mobile artifact.

On the other hand, categories such as “decoration” and “numerical system,” or “decoration” and “writing system” are not mutually exclusive. Rather, sign systems can be used as decoration without losing their information value. This is exemplified in historic times by calligraphy, inscriptions on pottery and temples, tattoos of graphemes on human bodies, and many other artistic expressions. “Information density” in an information-theoretic sense is a fundamental property of a sign sequence, irrespective of whether there is a human present to interpret it—or merely find it aesthetically pleasing.

### Types of Signs.

In the context of semiotics, signs are typically divided into indeces, icons, and symbols (60 p. 86). In a nutshell, an index is a sign which stands in a direct causal relation with the object it represents, while an icon represents an object by means of resemblance/similarity. Finally, a symbol is a sign with a conventional and arbitrary (i.e. noniconic) relation to the object.

Some of the signs of the Swabian Aurignacian are likely indices. For instance, notches applied to flutes for indicating where holes need to be cut out. In fact, these straddle upon the category of “utilitarian.” They are still included in our sample since in some cases they occur without any obvious function relating to flute production. There are also credible examples of icons. For instance, on realistically crafted figurines of felines and fish we find dots. These mimic patterns which were discernible by the Paleolithic viewer when observing actual cave lions and trout. Interestingly, however, we also find dots on one side of the ivory plaquette which bears a relief of a hybrid creature between human and lion—the so-called “Adorant”—on the other side (Fig. 1*A*). In this case, the dots seem abstracted away from their original iconic usage, as well as linearly organized in rows of similar lengths, potentially representing numerical information. This would constitute a rather symbolic usage.

### The Meaning of Paleolithic Signs.

Given the variable nature of meanings associated with signs in human cultures, it is not surprising that there is a multitude of hypotheses about the meanings of Paleolithic signs in general, and the Swabian Jura in particular. For instance, crosses found on mammoth and horse figurines have been interpreted as either simple reflections of fur, counting devices, or symbols of ritual killings (17, pp. 69–80). The latter are found also in association with hunter-gatherer cultures in historic times (61). Marshack (24) claimed that some markings on cave walls and mobile objects encode lunar calendars. Leroi-Gourhan, on the other hand, discusses the hypothesis of so-called “hunting tallies,” as well as similarities to ritual message sticks in the ethnographic record (39, pp. 188–190). The apparent similarity to historical message sticks is an interesting avenue for future research. The recent development of a database for Australian message sticks is a first step in this direction (62, 63).

Linking back to the discussion of early protocuneiform, Sauvet et al. (64, p. 556) identified a set of recurring Paleolithic signs which resemble ideographs in ancient Sumerian, Hittite, Egyptian, and Chinese protowriting. For instance, branch-shaped signs of the Paleolithic resemble ideographs in protowriting denoting “grain” or “herb.” However, “decipherment” of Paleolithic signs in a strict sense is severely hampered by the sheer time depth of this material.

Most recently, it was hypothesized that sign sequences associated with animals in cave art and on mobile objects across the Upper Paleolithic of Europe constitute “phenological calendars” (42). In this interpretation, “Y” signs denote “giving birth,” and sequences of lines or dots represent natural numbers reflecting the passage of time in lunar months. Such calendars would have helped ancient hunter-gatherers to store crucial information about the life cycles and yearly migrations of their prey. Some methodological criticisms of this particular account have been published recently (65–67).

While statistical analyses by themselves cannot conclusively answer questions about meanings of sign sequences, they can, at a minimum, help to narrow down the range of possible interpretations. Given samples of sequences for which we know the interpretation, we can test whether a set of unknown sequences displays a similar statistical fingerprint. The same statistical fingerprint constitutes a necessary but not sufficient condition for functional equivalence.

### The Question of Writing.

Whenever unknown signs are discovered in archaeological contexts, a natural first question to ask is whether they constitute “writing.” The standard philological definition of writing refers to the tight link between spoken language, on one hand, and the graphical marks representing it, on the other:

“Broadly defined, writing represents speech. One must be able to recover the spoken word, unambiguously, from a system of visible marks in order for those marks to be considered writing, [...]” (68, p. 18)

Our results strongly contradict the hypothesis that the sign sequences of the Swabian Aurignacian constitute writing in this strict sense. Their statistical properties are very different from those of genuine writing systems around the world. Rates of adjacent sign repetitions are generally very high in Aurignacian sequences, while they are generally very low in modern day writing systems. This is true regardless of whether we consider alphabets, syllabaries, or morpho-syllabaries (“logographies”). While in our sample there are occasional examples of adjacent character or morpheme repetitions in alphabetic writing (French *J’e**ss**aie* “I try”), and morpho-syllabic writing (e.g. adjacent repetition of the Hanzi character for the negation particle *bù* in Mandarin Chinese), these are far and few between.

It has been pointed out before that human languages exhibit repetition avoidance at multiple levels of structure (e.g. phonemes, morphemes, words) (69–71). In natural languages, the same structural elements are unlikely repeated in adjacency. This leads to higher information density (more information in a given time interval), potentially linked to cognitive biases of production and usage (71). Against this backdrop, it is very unlikely that the repetitive sign sequences on mobile artifacts represent the structure of languages spoken by the Aurignacian people of the Swabian Jura—the sole criterion for writing sensu strictu.

However, it is noteworthy that this strict definition of writing has been repeatedly called into question:

“This would mean that writing is, as some linguists assume, a device for the recording of speech and that all the stages in which writing does not serve this purpose are only feeble attempts in the direction of writing, but not real writing [...] This restriction of the definition of writing is unsatisfactory, however, because it does not take into account the fact that both stages have one identical aim: human intercommunication by means of conventional visible marks.” (72, p. 12)

The Aurignacian sign sequences meet this broader criterion. Namely, they were produced consistently by different individuals over many generations, and they were systematically employed more productively on figurines than on other types of objects (cf. Fig. 4). Also, some sign types have been preferentially used with certain object types (*SI Appendix*, Fig. S5). For example, crosses are some of the most frequently occurring signs. In our sample, however, they never occur on anthropomorph figurines, but rather on zoomorph figurines (especially horses and mammoths) as well as on tools. Inversely, dots occur most frequently on anthropomorph figurines and also zoomorph figurines (especially felines), but never on tools. Note that this cannot be due to material constraints, as both figurines and (some) tools are carved out of ivory, and have surfaces which allow for application of either crosses or dots. Hence, there is a deliberate choice behind adorning humans and felines (cave lions) with dots, but other animals like horse and mammoth as well as tools with crosses. The members of the Swabian Aurignacian culture have certainly handed down such conventions over generations of ivory carvers, otherwise the occurrence of these statistical patterns over 10,000 y would be extremely unlikely.

However, we do not want to conceal the fact that the human intercommunication criterion is rather loose, and also applies to traffic signs, license plates, Morse code, tattoos, message sticks, and many other devices storing information outside of human minds for intercommunication purposes. When talking about “writing” the strict definition is appropriate. It allows us to further subdivide the vast space of human sign systems into those which are “linguistic” vs. those which are “nonlinguistic” (73). In fact, this division is supported by the empirical evidence, as written languages carry a statistical fingerprint related to structural biases in spoken, and likely also signed languages (29).

In sum, the sign sequences of the Swabian Aurignacian might be described as human intercommunication by means of conventional visible marks, while they certainly do not meet the criterion of writing sensu strictu.

### Taxonomy of Sign Systems and Writing.

Writing in the strict sense—i.e. sign sequences structurally representing spoken language—has developed independently at least three to four times, namely, in Mesopotamia, Egypt, China, and Mesoamerica (68), and potentially a fifth time in the Eastern Pacific (31). These developments fall in the range of roughly 2500 BC to 1500 AD.

However, it has been argued before that the development of writing can only be understood against the backdrop of prehistoric sign systems (74, p. 89). This is underlined, for instance, by analyses of the so-called *La Marche* antler, likely deriving from the Upper Magdalenian period of western France—roughly 15,000 y ago. It is adorned with rows of different sign types which have been interpreted as part of a complex artificial memory system (37).

A possible direction for future research is to craft a taxonomy of sign systems from the Paleolithic to the modern day (73). This requires a set of measurable design features, such as the sign inventory size, linear arrangement, combinatoriality, conventionalization etc. Arguably, some of these design features are already present in the Aurignacian period of 40,000 y ago, e.g. sign inventory size and (to some extent) linear arrangement, while others are largely lacking, e.g. (adjacent) combinatoriality of different sign types, or cannot be strictly proven, e.g. the rebus principle linking sounds of spoken language to signs.

Such a taxonomy will enable a multidimensional comparison between different human sign systems of which writing is but one particular facet.

### Commonalities and Differences with Early Protocuneiform.

Our results illustrate that the statistical properties of sign sequences from the Swabian Aurignacian are very similar to those of the Uruk V protocuneiform period (roughly 3500 to 3350 BC). For the later protocuneiform periods of Uruk IV (roughly 3350 to 3200 BC) and Uruk III (roughly 3200 to 3000 BC), however, we already find a significant divergence toward higher information encoding potential.

In this context, it should be noted that the latter periods reflect the transition from mostly numeric to so-called numero-ideographic notations—with a considerable increase in the number of signs in general, and ideographic signs in particular. While the sign list for Uruk V features 47 signs of which 39 (83%) are numeric and only 8 (17%) are ideographic, the list for Uruk III (approximately 150 to 350 y later) features 838 signs, of which only 105 (13%) are numeric, and 733 (87%) are ideographic.

The signs on purely numeric tablets are typically ordered according to their values (75, p. 52). For example, the sign produced by sideways indent of a reed stylus and denoted as N01 might represent the number one, and the sign produced by a vertical indent of a reed stylus and denoted as N14 might represent the number 10. See the Uruk V tablet in Fig. 2*A* as an example. The numerical values represented by signs are inferred for later periods by interpreting the bundling of signs and the maximum number of repetitions of a given sign. However, note that such bundling and maximum numbers are not always consistent especially in the earliest protocuneiform tablets assigned to Uruk V. Namely, there are examples on tablets where the repetitions of alleged lower number signs transgress the maximum numbers expected (75, p. 51).

The few numero-ideographic protocuneiform tablets assigned to Uruk V typically feature number signs in combination with ideographic signs representing commodities (vessels with some content, sheep, grain, etc.). See as an example the Uruk V tablet in Fig. 2*B*. This carries the sequence

[1] $$\begin{array}{c} N14\;N14\;\_\;N01\;N01\;N01\;N01\;N01\;\_\;DUG∼b, \end{array}$$

where DUG∼b denotes an iconographic representation of a vessel of unknown content. Due to problems of assigning clear numeric values to these early number signs, it is not possible to give an exact translation of this sign sequence. The meaning of this sequence for us is approximately: “some number of vessels of unknown content.”

In comparison, for the material of the Swabian Aurignacian, there are no descendant sign systems which could be used to infer the exact functions of the earlier signs. In the case of particular artifacts like the “Adorant” (Fig. 1*A*), it has long been argued that these constitute deliberate numerical representations, as the sequences are bundled to similar lengths (10 to 13 dots vertically and 4 horizontally on the flat surface of the plaquette), and these lengths are not simply the outcome of the material constraints (76). In combination with the anthropomorph figurine, this would constitute a numero-ideographic representation. However, in contrast to protocuneiform, there is currently no evidence of higher numbers being encoded with separate, more complex signs.

Protocuneiform in Mesopotamia was exapted to reflect the Sumerian language morpho-phonetically by the Early Dynastic Fara period of approx. 2500 BC (75, p. 80)—i.e. around 1000 y after the earliest period of Uruk V numero-ideographic tablets. This process was fueled by the complexification of the economy [cf. 75, 77). The growing economy required, first, a drastic increase in sign types representing numerals, measures of time, and commodities, and second, an administrative apparatus of scribes. These scribes would gradually transform the signs from reduced depictions to abstract symbols via repetitive usage. This abstraction, in turn, helped to detach the signs from their original referents, and exapt them to represent the sounds of the Sumerian language instead (78).

In stark contrast, we do not detect any significant change in the sign repertoire or in the information density of sequences in the case of Swabian Aurignacian mobile artifacts over the course of 10,000 y.

### The Cognitive Implications.

Our findings relate to the hypothesis that human intelligence arose through “expanded information capacity” (79). Namely, from an information-theoretic perspective, producing the Aurignacian sequences arguably required roughly as much “information capacity” as the earliest protocuneiform (Uruk V), but clearly less than later protocuneiform periods (Uruk IV and Uruk III) as well as writing systems around the world. This has to be seen against the backdrop of theoretical results showing that the entropy is an upper bound on the mutual information between signs and referents (80). In other words, the entropy of a sign system is a measurable restriction reflecting the potential to encode sign/referent mappings unambiguously. Our analyses hence suggest that the first hunter-gatherers arriving in Central Europe more than 40,000 y ago already had the information capacity to create a sign system comparable to protocuneiform in terms of information encoding potential.

## Conclusions

In conclusion, the people of the Swabian Aurignacian were among the first modern humans to settle in Central Europe. They inhabited the caves of the Lone and Ach Valleys around 43,000 to 34,000 calibrated years ago. They have left behind a panoply of tools and mobile artwork—testimony to their technical skills, and their rich culture. This includes a collection of several dozen ivory figurines, representing animals occurring in the environment at the time, as well as mystical figures of an imaginary world forever lost to us. A subset of these mobile artifacts carry sequences composed of overall more than 3000 signs intentionally carved into their surfaces.

Our analyses show that these sequences are clearly statistically distinct from those generated with modern day writing systems to represent spoken languages. However, they have a very similar “statistical fingerprint” as the earliest numeric and numero-ideographic protocuneiform tablets stemming from the Uruk V period of 3500 to 3350 BC. Moreover, the Aurignacian sequences were not indiscriminately applied to different objects, rather, ivory figurines carry the sequences of highest information density—independent of material constraints such as volume and preservation. The hunter-gatherers of the Swabian Aurignacian have hence developed a sign system with some incipient design features also found in writing, that is, an inventory of different sign types and their linear arrangement, but lacking other design features, e.g. productive combinatoriality of different sign types as well as the rebus principle.

It remains hard—or impossible—to prove that Aurignacian sign systems served the same numero-ideographic functions as protocuneiform. Moreover, there is another stark contrast between them: Protocuneiform developed into a full-blown writing system representing the Sumerian language within the subsequent 1,000 y. The sign sequences of the Swabian Aurignacian, on the other hand, were stable in terms of information density—for 10,000 y—and then disappear.

## Materials and Methods

The corpus for the current analyses consists of overall 260 artifacts (213 after preprocessing) carrying signs for the caves of the Swabian Jura. Most such objects derive from Vogelherd Cave in the Lone Valley and Hohle Fels Cave in the Ach Valley. The most common (identifiable) object types carrying signs are tools, zoomorph figurines, tubes and/or flutes, and personal ornaments. The vast majority of these mobile objects is made of mammoth ivory, followed by bone, and antler. An overview of these statistics is given in *SI Appendix*, Fig. S6. Drawings of objects and the identified sign sequences are made available via the SignBase website (https://www.signbase.org/) (5). The sign types and sequence codings are also further explained at https://www.signbase.org/description/ under the point “sign coding.” A table with up-to-date UTF-8 definitions of sign types can be downloaded at https://www.signbase.org/download/.

### Protocuneiform (Uruk).

Information on protocuneiform tablets is taken from the Cuneiform Digital Library Initiative (CDLI) (81). This online database provides pictures, meta-data, transliterations, and standardized sign lists for automated processing. In our analyses, we include transliterations for the Uruk V (roughly 3500 to 3350 BC), Uruk IV (roughly 3350 to 3200 BC), and Uruk III (roughly 3200 to 3000 BC) periods. For some example tablets see Fig. 2. Note that the exact dating of these periods—and the attribution of particular tablets to these—is complicated by the occurrence of so-called secondary deposits (75), and more general problems relating to the organization of excavation campaigns (82). Regardless of their exact dating, however, the respective tablets are commonly discussed as the precursors of cuneiform writing (74, 75, 83, 84). For the earliest period of Uruk V, the number of available transliterations is ca. 100, while for the latter two there are several thousand. In order to not heavily overrepresent the latter in the analyses, we take subsamples of 1,000 for each.

### Modern Written Languages.

Modern day languages are represented by the Text Data Diversity sample (TeDDi) (59). It features 89 languages (according to ISO 639-3 codes) of 58 different language families, written in 16 different scripts (according to ISO 15924 codes). Again, 1,000 lines of linguistic utterances are randomly sampled from the TeDDi sample to roughly match the number of sequences available also for protocuneiform periods, and the Aurignacian material. This subsample includes 33 languages and 14 different scripts. See *SI Appendix*, Fig. S7 and Table. 1 for some example sequences.

**Table 1.** Examples of sequences and feature values

Subcorpus	Sequence	#$t^{\prime }$	#*t*	$\hat{H}$	$\hat{h}$	TTR	*r*
TeDDi (Basque)	Mila esker maitea	17	11	3.29	2.19	0.65	0
TeDDi (Chinese)	see *SI Appendix*, Fig. S7 ID13125567	7	6	2.52	1.69	0.86	0.17
TeDDi (Chinese strokes)	etao etao aieeeaetn eaaieeeeto taieetso oodtshn etasee	54	10	2.89	1.99	0.19	0.17
Uruk III	N01 _ NAGA∼a _ DU _ PAP∼a	7	5	2.13	1.43	0.71	0
Uruk IV	N14 N14 _ N01 N01 N01 _ UDU∼a	8	4	1.91	1.37	0.5	0.43
Uruk V	N01 N01 N01	3	1	0	0.43	0.33	1
Aurignacian	XX_vvvvvvvv_vvvv	16	3	1.06	1.04	0.19	0.73

Selected sequences with number of sign tokens ($t^{\prime }$), number of sign types (*t*), as well as type-token ratios (TTR), estimated unigram entropies ($\hat{H}$), entropy rates ($\hat{h}$), and repetition rates (*r*).

#### Preprocessing.

All sequences are coded in UTF-8 characters. For a visualization of the preprocessing procedure see *SI Appendix*, Fig. S7. For natural languages (TeDDi sample) punctuation is removed, as well as characters which are generally not part of the respective writing system. A single “sign” here corresponds to a UTF-8 character (including white spaces)—compare also sign counts under *#Tokens* in *SI Appendix*, Fig. S7.

In the case of protocuneiform, annotations of the original transliterations in CDLI are removed. Also, the numerical notation to indicate repetitions of the same sign types are automatically expanded to yield strings of sign tokens, e.g. 3(NO1) → NO1 NO1 NO1. Note that NO1 stands for a single indent with a stylus on the clay tablet (often representing the number one). Underscores are added between visual groups of signs. Note that a “sign” here corresponds to a string of characters in the Latin transliteration (e.g. NO1, N14, UDU∼a).

In the case of Aurignacian objects, sign sequences are cleaned by a) removing annotations (e.g. “!” indicating broken pieces), b) removing signs which are not visually discrete (marked by round brackets), and c) removing signs which are not in linear order (marked by square brackets). Underscores indicate that the respective sign strings are on different parts of an object. Signs are here represented by UTF-8 characters, e.g. “|:” straight line, “v:” notch, “X:” cross, etc. For the full typology of sign types, see www.signbase.org. In *SI Appendix*, Fig. S5, we give frequency distributions of sign types by cave site, material, and object type.

#### Statistical features.

Given the preprocessed sequences of Aurignacian signs, protocuneiform writing, and modern writing, their “statistical fingerprint” is established, i.e. quantitative features which have been proposed to distinguish natural languages from other sequences (25–27, 29, 85). In particular, we harness the type-token ratio (TTR), the unigram entropy (*H*), the entropy rate (*h*), as well as the repetition rate of adjacent signs (*r*).

Let us assume a vocabulary of sign types $V=\left\{t_{1},t_{2},\cdots ,t_{m}\right\}$, with m∈N being the finite size or cardinality of the set, i.e. m=|V|. Sign types are unique, i.e. $t_{1}\neq t_{2}\cdots \neq t_{m}$. A sequence is then a concatenation of sign tokens ($t_{i}^{\prime }$) such that

[2] $$\begin{array}{c} s=(t_{1}^{\prime },t_{2}^{\prime },\cdots ,t_{N}^{\prime }), \end{array}$$

where n∈N is the length of the sequence in tokens. Note that sign tokens are not unique, i.e. it is possible that $t_{1}^{\prime }=t_{2}^{\prime }\cdots =t_{n}^{\prime }$ for some type $t_{i}$. In the following, *m* and *n* represent the vocabulary sizes and lengths of particular sequences (i.e. $m_{s}$ and $n_{s}$ in a strict sense). The s is dropped for simplicity.

### Type-Token-Ratio (TTR).

The type-token ratio of a given sequence s is then defined as

[3] $$\begin{array}{c} \text{TTR}\left(s\right)=\frac{\left|V\right|}{\sum_{i=1}^{\left|V\right|}f_{i}}=\frac{m}{n}, \end{array}$$

with $f_{i}$ being the absolute token frequency count in the sequence s for a given type $t_{i}$. In words, the number of different types in a sequence (*m*) is divided by the number of tokens (*n*). The TTR is a simple, well-known, all-purpose quantitative linguistics measure, which can be seen as a baseline. It reflects the diversity of sign types given the overall number of sign tokens. It is in the range (0,1], if we only consider nonempty sequences, i.e. 0<m≤n. For example, in the Aurignacian sequence XX_vvvvvvvv_vvvv (see vhc0096 in *SI Appendix*, Fig. S7) the “vocabulary” is the set $V=\left\{X,v,\_\right\}$, and the token frequencies of sign types are “X:” 2, “v:” 12, “_:” 2. We thus get TTR=3/16∼0.19. Compare also Table. 1.

### Unigram Entropy (*H*).

Compared to TTR, the Shannon entropy of unigrams is a more nuanced measure of diversity, reflecting the probability distribution over the elements of the vocabulary (86, pp. 51–55). Let us assume a discrete random variable *S* which takes the vocabulary V as its support, that is, each draw will result in a token representing a type $t_{i}\in V$. The probabilities of types are represented by the probability mass function p(t)=P(S=t). The entropy of *S* is then theoretically defined as (87, p. 14)

[4] $$\begin{array}{c} H\left(S\right)=-\sum_{t\in V}p\left(t\right)\log_{2}p(t) \end{array}$$

In fact, it can be seen as an average over the information content of types which is given by

[5] $$\begin{array}{c} I\left(t\right)=-\log_{2}p(t). \end{array}$$

This takes values in the range [0,∞). For a type of maximal probability, i.e. $p(t_{i})=1$, we have $-\log_{2}\left(1\right)=0$, and for a type of minimal probability, i.e. $p(t_{i})=0$, we have $-\log_{2}\left(0\right)=\infty$. The original rationale of Claude E. Shannon (88) is that likely events provide us with less information than unlikely events. The entropy is the average information content of a vocabulary of types, hence also falling theoretically in the range [0,∞). However, for a finite sequence s, the maximal entropy is empirically upper-bounded by the case of uniform probability over types in V, i.e. $p\left(t\right)=\frac{1}{m}$, yielding

[6] $$\begin{array}{c} \hat{H}\left(s\right)\leq -\sum_{t\in V}\frac{1}{m}\log_{2}\frac{1}{m}\equiv \log_{2}\left(m\right). \end{array}$$

Again, this property was deliberately chosen by Shannon (88) to reflect the fact that maximal uncertainty should be given in the case all events are equally likely to occur.

A crucial problem for entropic investigations of finite sequences is how to estimate the probabilities p(t) of types. We here employ the so-called maximum likelihood (ML), or plug-in estimator

[7] $$\begin{array}{c} \hat{p}{\left(t_{i}\right)}^{\text{ML}}=\frac{f_{i}}{\sum_{i=1}^{\left|V\right|}f_{i}}=\frac{f_{i}}{n}, \end{array}$$

where $f_{i}$ is, as above, the absolute frequency of a type $t_{i}$ in the sequence s. The probability of a given type is hence seen as a relative frequency over the length of the sequence. Plugging Eq. **7** into Eq. **4** gives the maximum likelihood estimated unigram entropy of a sequence. We call this the unigram entropy (${\hat{H}}^{\text{ML}}$). For example, for the preprocessed Mandarin Chinese sequence in *SI Appendix*, Fig. S7 (ID13125567) we have

[8] $$\begin{array}{c} \begin{array}{c} {\hat{H}}^{\text{ML}}\left(s\right)=-\left(\frac{2}{7}\log_{2}\left(\frac{2}{7}\right)+\frac{1}{7}\log_{2}\left(\frac{1}{7}\right)+\cdots +\frac{1}{7}\log_{2}\left(\frac{1}{7}\right)\right)\\ ∼2.89\;\text{bits/sign}. \end{array} \end{array}$$

There is a range of further entropy estimators which have been developed to alleviate data sparsity problems by smoothing probabilities. However, it turns out that all of them are highly correlated for language data (86).

### Entropy Rate (*h*).

While the TTR and the unigram entropy *H* only take into account the frequencies/probabilities of individual types—independent of their cotext—the concept of entropy rate can be employed to assess the predictability given that signs in a sequence might display systematic co-occurrence patterns.

Formally, instead of a single random variable *S* we consider a stochastic process $S=\{S_{1},S_{2},\cdots ,S_{n}\}$ with vocabulary V. This can be seen as a concatenation of random variables, rather than a single random variable. In theory, the entropy rate for a stochastic process is defined as (87, p. 74)

[9] $$\begin{array}{c} h\left(S\right)=\lim_{n\to \infty }\frac{1}{n}H\left(S_{1},S_{2},\cdots ,S_{n}\right), \end{array}$$

With $H(S_{1},S_{2},\cdots ,S_{n})$ representing the joint entropy of the random variables. Thus, the entropy rate is only defined in the limit of a sequence with infinite length. Given a finite sequence s, Gao et al. (89) equation (6) propose an increasing-window estimator based on Lempel-Ziv compression (LZ78). This is defined as

[10] $$\begin{array}{c} \hat{h}\left(s\right)=\frac{1}{n}\sum_{i=2}^{n}\frac{\log_{2}\left(i\right)}{L_{i}}, \end{array}$$

where *i* is a given position in the sequence $s=(s_{1},s_{2},\cdots ,s_{n})$, and $L_{i}$ is the length (+1) of the longest contiguous subsequence starting at position *i* which is also present in the so-called “prefix,” i.e. in the subsequence $s_{1}^{i-1}$. Note that the numerator $\log_{2}\left(i\right)$ gives the maximally possible entropy of the sequence up to position *i*, namely, when we have seen only unique types such that i=m (remember Eq. **6**). The denominator, on the other hand, is a representation of the repetitiveness of the sequence. Take the sequences $s_{1}=\left(same\_but\_different\right)$ and $s_{2}=\left(same\_same\right)$ as an example. In position i=6, for both we have $\log_{2}\left(6\right)∼2.58\;\text{bits/sign}$ in the numerator. However, for $s_{1}$ we have $L_{6}=0+1=1$, while for $s_{2}$ we have $L_{6}=4+1=5$ in the denominator. Overall the ratio is then $\frac{2.58}{1}∼2.58\;\text{bits/sign}$ for $s_{1}$, and $\frac{2.58}{5}∼0.52\;\text{bits/sign}$ for $s_{2}$. In other words, the repetitiveness inherent to $s_{2}$ is penalized when estimating the entropy rate.

One problem with this estimator is that it converges relatively slowly for highly repetitive sequences. This has to do with the fact that as we approach positions toward the end of the sequence, the $L_{i}$s necessarily become shorter since the subsequences after position *i*, i.e. $s_{i}^{n}$, become shorter. This leads to an overestimation bias. See also simulations in Gao et al. (89, p. 91). We illustrate the stabilization properties for our specific dataset in *SI Appendix*, Figs. S9–S12.

### Repetition Rate (*r*).

The repetition rate has been proposed by Richard Sproat (27) as a feature to distinguish writing from nonwriting. It measures the number of adjacent repetitions of signs in a sequence over the number of possible repetitions. Formally, it can be defined as

[11] $$\begin{array}{c} r^{\text{sproat}}\left(s\right)=\frac{r^{\text{adj}}}{\sum_{i=1}^{m}\left(f_{i}-1\right)}, \end{array}$$

where $r^{\text{adj}}$ in the numerator stands for the number of adjacent repetitions of sign types. More precisely, we have a repetition if for two tokens in the sequence s it holds that $t_{j}^{\prime }=t_{j+1}^{\prime }=t_{i}$. The denominator is a normalization given the possible number of repetitions in the sequence, where $f_{i}$ is the token frequency of a type $t_{i}$ and *m* is the overall number of types—as above. This normalization term is named *R* in the original publication (27). Note that for any given type the maximum possible number of adjacent repetitions is $f_{i}-1$. In Sproat’s definition, this is summed up over all types to get *R*.

We here slightly modify this definition by changing the denominator to n−1 instead, i.e. simply the length of the sequence s minus one (abbreviated to *lsmo* here). We thus have

[12] $$\begin{array}{c} r^{\text{lsmo}}\left(s\right)=\frac{r^{\text{adj}}}{n-1}=\frac{r^{\text{adj}}}{(\sum_{i=1}^{m}f_{i})-1} \end{array}$$

Both versions of the repetition rate *r* take values in the range [0,1]. The main difference is that for sequences with many unique types $r^{\text{sproat}}$ will give high values, while $r^{\text{lsmo}}$ will give low values. For example, for the sequences (aaaaa) and (aabcd), we have $r^{\text{sproat}}=1$ in both cases, while we have $r^{\text{lsmo}}=1$ and $r^{\text{lsmo}}=0.25$ respectively.

We have implemented both versions of the repetition rate in the statistical software R (90). The function is called rrate(). We generally use the *lsmo* version in this study.

Further details on the convergence properties of the statistical features as well as randomization analyses are given in *SI Appendix*.

#### Classification algorithms.

Given the feature value estimations, for each sequence s we get a four-dimensional vector ($\vec{v}$) of values. In other words, a sequence is here represented as a point in a four dimensional space, i.e.

[13] $$\begin{array}{c} {\vec{x}}_{s}=\left[\begin{array}{c} x_{1}=\hat{H}\left(s\right)\\ x_{2}=\hat{h}\left(s\right)\\ x_{3}=\;\text{TTR}\left(s\right)\\ x_{4}=r\left(s\right) \end{array}\right] \end{array}$$

For example, for the Basque, the Mandarin Chinese, and the Aurignacian sequences in Table. 1, we have

[14] $$\begin{array}{c} {\vec{x}}_{s^{\text{basque}}}=\left[\begin{array}{c} 3.29\\ 2.19\\ 0.65\\ 0 \end{array}\right],{\vec{x}}_{s^{\text{mandarin}}}=\left[\begin{array}{c} 2.52\\ 1.69\\ 0.86\\ 0.17 \end{array}\right],{\vec{x}}_{s^{\text{aur}}}=\left[\begin{array}{c} 1.06\\ 1.04\\ 0.19\\ 0.73 \end{array}\right]. \end{array}$$

A core question in this research is whether it is possible to clearly classify these sequences based on their feature values. This boils down to a standard classification task: Can we assign the correct labels, i.e. names of subcorpora (TeDDi, UrukIII, UrukIV, UrukV, Aurignacian), to the sequences based on their feature values? If yes, then the statistical fingerprint of sequences can indeed be used to identify the subcorpus they belong to.

### Training and Test Sets.

In total, we have 3078 sequences with feature value estimations and subcorpus labels. Note that we always test classification performance on pairs of subcorpora (i.e. Aurignacian vs. UrukV, Aurignacian vs. UrukIII, Aurignacian vs. UrukIV, and Aurignacian vs. TeDDi). These pairwise subsamples are then split into training and test sets by the ratio 67% to 33%. An overview of the subsample structure is given in Table. 2.

**Table 2.** Number of sequences for subcorpus pairs overall, training and test sets

Subcorpus1	Subcorpus2	Overall	Training	Test
Aurignacian	TeDDi	1,253	850	403
Aurignacian	UrukIII	1,068	728	340
Aurignacian	UrukIV	1,072	732	340
Aurignacian	UrukV	324	216	108

These training and test sets of sequences are used as input to classification algorithms. We use K-Nearest-Neighbors (KNN) as a baseline classification algorithm, and Multilayer Perceptrons (MLP), also known as deep feedforward neural networks, as state-of-the-art classification algorithms.

With regard to neural network architectures, i.e. hidden layer depth and size, we randomly sample 100 architectures with a maximum depth of four layers and a maximum size of five hidden units per layer. More complex architectures are unlikely to converge with our dataset. An example of a complex architecture which converges is given in *SI Appendix*, Fig. S13.

In summary, for each binary classification between subcorpora we have theoretically up to 100 results—one for each architecture. In practice, there are fewer results, since not all architectures converge. The neural net analyses were run on a 16 GB NVIDIA RTX A4000 GPU. Further mathematical details on the classification algorithms are given in *SI Appendix*.

### Performance Statistics.

The accuracy, precision, recall, and F1 scores are reported for each classification run—corresponding to one value of *k* for a KNN, or one MLP architecture trained and tested on the sequences.

We here focus on the accuracy as this comes with a straightforward baseline and a standard statistical test for significant deviation from the baseline. The accuracy is given as the number of correct labels assigned to sequences of the test set (successes), over the total number of labels assigned (successes plus failures). A worked example of how to calculate the test statistic is given in *SI Appendix*.

For each *k* in KNN classification, and for each model architecture in MLP classification, we get a *P*-value reflecting whether the success rate of the given classification algorithm is significantly better than the baseline. Since we run many statistical tests, we additionally correct the *P*-values by the Bonferroni method, i.e. multiplying the *P*-value by the number of tests while keeping the *α*-level constant.

If the sequences have different statistical properties, then they should be distinguishable by the classification algorithms, and there should be many *P*-values below the *α*-level. We provide the percentage of significant *P*-values alongside the distributions of accuracy values in *SI Appendix*, Fig. S4.

#### Statistical models for Aurignacian data.

Besides the classification analyses, we also provide regression models for the Aurignacian data to further contextualize the usage of sign sequences by ancient humans of the Swabian Jura. In particular, we are interested which meta-data categories per object predict higher or lower information density of the sequences on it.

### Response Variable.

We use the entropy rate as response variable, since this feature reflects both the diversity of the sign types used on an object (similar to TTR and unigram entropy), but also their sequential structure (similar to the repetition rate). However, the R code is implemented such that the statistical feature can be chosen in a single line of code, and all the analyses can be rerun without changing the rest of the code. Furthermore, note that the entropy rate is correlated with the number of sign tokens and the number of sign types per sequence. However, using these as response variables will lead to violation of regression assumptions (e.g. normality of residuals).

### Predictor Variables.

As predictor variables we consider the object type (factor variable: “anthropomorph figurine,” “zoomorph figurine,” etc.), material (factor variable: “antler,” “ivory,” etc.), cave site (factor variable: “Hohlenstein-Stadel,” “Hohle Fels,” etc.), preservation (factor variable: “almost complete,” “fragmented,” “complete”), volume in cm^3^ (continuous variable), and maximum estimated age of the object (approx. 40,000 BP to 30,000 BP). See also *SI Appendix*, Fig. S14.

### Model Formulation.

Given the response variable $Y_{\text{hrate}}$ we first define a baseline model with a single parameter $\beta_{0}$, i.e., the intercept or average of *Y* such that $\beta_{0}=\bar{Y}$:

[15] $$\begin{array}{c} \begin{array}{c} Y_{\text{hrate}}=\beta_{0}+\epsilon ,\\ \epsilon ∼N(0,\sigma_{\epsilon }^{2}). \end{array} \end{array}$$

The errors *ϵ* (or residuals) are assumed to be normally distributed around this mean. We then build models stepwise by adding theoretically relevant predictors one at a time, following the advice in Baayen (91), and comparing the respective model to the former model by means of AIC—with lower AIC indicating a preferable model (Table. 3). For example, with the continuous predictor “object volume” ($X_{1}$) added we have

[16] $$\begin{array}{c} \begin{array}{c} Y_{\text{hrate}}=\beta_{0}+\beta_{1}X_{1}+\epsilon ,\\ \epsilon ∼N(0,\sigma_{\epsilon }^{2}), \end{array} \end{array}$$

**Table 3.** Multiple regression model results

Model specification	DF	r. DF	F	*P*	AIC
Intercept	1	212	NA	NA	198
Intercept + volume	2	207	23	2e-06	175
Intercept + volume + date	3	203	14	2e-06	171
Intercept + volume + date + object_type	8	198	10	2e-10	145
Intercept + volume + date + object_type + site_name	10	196	8	5e-10	147
**Intercept + volume + date + object_type + preservation**	**10**	**196**	**8.5**	**1e-10**	**144**
Intercept + volume + date + object_type + material	11	195	7.2	1e-09	148
Intercept + volume*preservation + date + object_type	12	194	7	5e-10	146
Intercept + volume*object_type + date	13	193	6.5	1e-09	147
Intercept + volume + date*object_type	13	193	6.3	2e-09	149

The model specification gives the predictor variables included in the model. DF: degrees of freedom. r. DF: residual degrees of freedom (number of data points minus parameters). F: F-statistic. *P*: *P*-value for the F-test. AIC: Akaike Information Criterion. The best model in terms of AIC is highlighted in bold.

where $\beta_{1}$ is the coefficient which reflects the effect that the predictor variable has on the entropy rate. The best model arrived at in terms of AIC includes volume and date as continuous predictors, as well as object type (six levels) and preservation (three levels) as categorical predictors. Given the treatment dummy coding for covariance analyses in R (91, p. 339) this requires overall ten parameters (nine coefficients plus intercept) to be estimated:

[17] $$\begin{array}{c} \begin{array}{c} Y_{\text{hrate}}=\beta_{0}X_{0}+\beta_{1}X_{1}+\beta_{2}X_{2}++\beta_{3}X_{3}+\cdots +\beta_{9}X_{9}+\epsilon ,\\ \epsilon ∼N(0,\sigma_{\epsilon }^{2}), \end{array} \end{array}$$

where $\beta_{0}$ is the group mean of *Y* for the data points with the two default factor levels, and $X_{0}$ is a variable always taking the value one, $\beta_{1}$ and $X_{1}$ as well as $\beta_{2}$ and $X_{2}$ might represent the effects of the continuous variables (volume and date) on *Y*, and the other coefficients $\beta_{3},\cdots ,\beta_{9}$ would then represent the effects of the binary dummy coded variables representing the levels of the categorical predictors $X_{3},\cdots ,X_{9}$. For this best model, all model assumptions (normality of residuals, homoscedasticity, etc.) are checked in *SI Appendix*, Fig. S15.

## Supplementary Material

Appendix 01 (PDF)

## Data Availability

The analyses are implemented in the statistical software R (90). The data of SignBase Version 2.0 is available at Zenodo: https://doi.org/10.5281/zenodo.18401937 (92). Data and R code of this study are available at Zenodo: https://doi.org/10.5281/zenodo.17673743 (93). This gives the permanent version of the github repository: https://github.com/christianbentz/PaleoSigns.
