# Supplementary material for: Humans 40,000 y ago developed a system of conventional signs
Source: Proc Natl Acad Sci U S A. 2026 Feb 23;123(9):e2520385123. doi: 10.1073/pnas.2520385123 (PMC12956821; doi:10.1073/pnas.2520385123)
Supplement: Supplementary file 1 — Appendix 01 (PDF) [file pnas.2520385123.sapp.pdf]

Supporting Information for

Humans 40,000 years ago developed a system of conventional signs

Christian Bentz, Ewa Dutkiewicz

Corresponding author: Christian Bentz.
E-mail: chris@christianbentz.de

This PDF file includes:

Figs. S1 to S15
SI References

Supplementary Methods

Feature estimation.

Convergence of feature values. Quantitative features such as entropies, TTRs, and repetition rates have different convergence properties, depending also on the estimators used. To assess the behaviour of estimated feature values with growing number of tokens, we give stabilization plots in Supplementary Figures S8-S11. It is visible in these plots that feature values start to stabilize at around 10-20 tokens for entropic measures, while the repetition rate is mostly stable right from the first tokens, and the TTR keeps decreasing with tokens even beyond 20. In the code files for subsequent analyses, we provide an option to exclude sequences of certain lengths to test the robustness of results.

Correlations between feature values. The four features are more or less correlated with one another. We give pairwise correlation plots also with the number of sign tokens (i.e. sequence length), and the number of sign types in Supplementary Figure S3.

Randomization. In the case of Aurignacian sign sequences, we do not know the order in which strings of signs were arranged. In very few cases can the order of application by an ivory worker be inferred. We therefore randomize the sign strings (delimited by underscores) within the sequences and compare the results for our original coding with the randomized one. Note that randomization can only affect the entropy rate, since TTR, unigram entropy, and the repetition rate of individual characters are independent of the order of sign strings. In Supplementary Figure S12, we show that entropy rates of the original and randomized sequences are almost perfectly correlated ($r = 0.99$, $p < 0.0001$) and can hence be interchanged without changing the results.

Classification.

K-Nearest Neighbors (KNN). KNN is a non-parametric and fast classification algorithm which has been around in machine learning since the mid 20th century (1). For general classification problems it is still competitive today – see leader board at https://paperswithcode.com/task/classification (last accessed 15/08/2024). The KNN algorithm is strictly speaking not "learning" from the training set, rather, it simply computes Euclidean distances for each vector of feature values in the test set with each vector of feature values in the training set. For two vectors of four dimensions the Euclidean distance is defined as

$$d(\vec{u}, \vec{v}) = \sqrt{(u_1 - v_1)^2 + (u_2 - v_2)^2 + (u_3 - v_3)^2 + (u_4 - v_4)^2}. \quad [1]$$

For example, the Euclidean distance between two of the Basque and Mandarin vectors is

$$d(\vec{x}_{\mathbf{s}^{\text{basque}}}, \vec{x}_{\mathbf{s}^{\text{mandarin}}}) = \sqrt{(3.29 - 2.52)^2 + \cdots + (0 - 0.17)^2} \sim 0.96. \quad [2]$$

For comparison, the Euclidean distance between the Basque and Aurignacian example vectors is

$$d(\vec{x}_{\mathbf{s}^{\text{basque}}}, \vec{x}_{\mathbf{s}^{\text{aur}}}) = \sqrt{(3.29 - 1.06)^2 + \cdots + (0 - 0.73)^2} \sim 2.65. \quad [3]$$

Hence, the Basque and Mandarin sequences are considerably closer in feature space than the Basque and Aurignacian sequences. If we imagine that only the Basque sequence was in the test set, and only the Mandarin and Aurignacian sequences in the training set, then the KNN algorithm would in this case correctly assign the label of the Mandarin sequence, i.e. "TeDDi", to the Basque sequence. More generally, KNN classifies a given target vector in the test set based on a majority vote of the class labels of the k neighbors (in the training set) nearest to the target vector. The number of nearest neighbors k taken into consideration is then the only hyperparameter to tune in the classification analyses. In our case, we consider k in the range $k = 1$ to $k = 10$. For each binary classification between subcorpora we hence have ten results.

Multi-Layer Perceptrons (MLP). Multilayer perceptrons (deep feedforward networks) have been the workhorses of deep learning in the last two decades (2, 3). They are still implemented as components of much more complex models, such as the original transformer architecture (4). The simplest feedforward architecture imaginable for binary classification with our data would feature four input units (one for each component of the feature vector), a single hidden unit, and a single output unit.

In this simple case, the activation in the single hidden unit corresponds to

$$h_1^{(1)} = f(\sum_{i=1}^{n=4} w_{1,i} x_i + b_1^{(1)}), \quad [4]$$

where the $w_{1,i}$s are the weights given to the input feature components, and $b_1^{(1)}$ is a bias added to the linear combination of input components. Note that the weights are here equivalent to coefficients in a regression model, and the bias corresponds to an intercept. Furthermore, a non-linear activation function $f()$ is applied to the linear combination. We arrive at the estimated

output value $\hat{y}$ by multiplying with another weight (connection between the hidden layer and the output layer), adding another bias, and finally, by applying another non-linear activation function $g()$.*

$$\hat{y} = g(f(\sum_{i=1}^{n=4} w_{1,i}x_i + b_1^{(1)})w_{2,1} + b_1^{(2)}), \quad [5]$$

In the case of binary classification, the output $\hat{y}$ is transformed to a probability in the range $[0, 1]$ to predict the correct classification label given the feature value input vector, i.e.

$$\hat{y} = P(y = 1|\vec{x}), \quad [6]$$

where $y = 1$ would correspond to one of the two possible classification labels, e.g. "Aurignacian" vs. "UrukV". In our particular setup, we have the input feature value vectors $\vec{x}_1, \vec{x}_2, \ldots, \vec{x}_n$ and respective class labels $y_1, y_2, \ldots, y_n$, where n corresponds to the size of the training set. These are used to learn the optimal weights and biases, i.e. the set of parameter values

$$\Theta = \{w_{1,1}, w_{1,2}, w_{1,3}, w_{1,4}, w_{2,1}, b_1^{(1)}, b_1^{(2)}\}. \quad [7]$$

"Optimal" here means these parameter values minimize the cost (i.e. error) over all pairs of estimated $\hat{y}_j$ and observed y_j output labels. For further details on feedforward neural network architectures, as well as the backpropagation algorithm for learning optimal parameters see Goodfellow et al. (5, p. 168). It has been proven that deep feedforward networks are in principle capable of representing any functional mapping between input and output units given enough hidden units (5, p. 198-199). However, in comparison to KNNs, MLPs are considerably harder to apply since they feature several hyperparameters which require fine-tuning for optimal results. These include the hidden layer architecture, the activation function, the error function, and the respective backpropagation algorithm (6–8), among others. We here use the hyperparameter settings which were shown to be most effective for a similar dataset and task in a pilot study on classifying writing and non-writing sequences (9).

Performance statistics. The accuracy, precision, recall, and F1 scores are reported for each classification run – corresponding to one value of k for a KNN, or one MLP architecture trained and tested on the sequences. We here focus on the accuracy as this comes with a straightforward baseline and a standard statistical test for significant deviation from the baseline.

For example, for the classification of Aurignacian vs. Uruk V proto-cuneiform, there are 108 sequences in the test set, of which 70 are Aurignacian, and 38 are Uruk V. Thus, the baseline in this case is

$$\frac{70}{108} = 0.648148148. \quad [8]$$

With a one-sided binomial test we can compute a p-value for whether the accuracy achieved by the model is significantly higher than the baseline (10, p. 15). Assuming a binomial distribution for random draws of two outcomes (e.g. 0 and 1 typically, or class labels UrukV and Aurignacian in our case) we have the null hypothesis and alternative hypothesis given as

$$H_0 : p = p_0 \quad [9]$$

and

$$H_1 : p > p_0 \quad [10]$$

respectively (11, pp. 11). Here p_0 represents the "no information rate" baseline, and p is the probability – or success rate – at which a given model identifies the correct class label. In the example given earlier, we have 108 sequences, i.e. $n = 108$ trials of classification. The expected value (mean) of successes (here termed B) under the null hypothesis ($p_0 = 0.648148148$) is

$$E_{p0}(B) = n \times p_0 = 108 \times 0.648148148 = 70, \quad [11]$$

and the variance is

$$\sigma_{p0}^2(B) = n \times p_0 \times (1 - p_0) = \\ 108 \times 0.648148148 \times 0.351851852 = 24.629629634. \quad [12]$$

The standardized version of B is then

$$B^* = \frac{B - E_{p0}(B)}{\sqrt{\sigma_{p0}^2(B)}}. \quad [13]$$

Under H_0 the standardized B^* is assumed to follow a normal binomial distribution $\mathcal{N}(0, 1)$. We can then identify a z-score given an α-level of our choice, which the actual B^* we calculate from the data would have to surpass to reject H_0. With the common $\alpha = 0.05$, we have $z_\alpha = 1.645$.

As a worked example, assume that in one of the runs of the MLP architecture classifying Aurignacian and Uruk V sequences the success rate is 75/108. In this case, we have:

$$B^* = \frac{B - E_{p0}(B)}{\sqrt{\sigma_{p0}^2(B)}} = \frac{75 - 70}{\sqrt{24.629629634}} \sim 1.0075 \quad [14]$$

Hence, $B^* < z_\alpha$, and we would accept H_0, meaning that the MLP classification is not significantly better than the "no information rate" baseline. The model has not learned (from the training set) to distinguish between these sequences better than the baseline (i.e. if it just chose the most frequent label).

*In our implementation, the activation function for hidden units corresponds to function for the output unit, i.e. $f() = g()$.

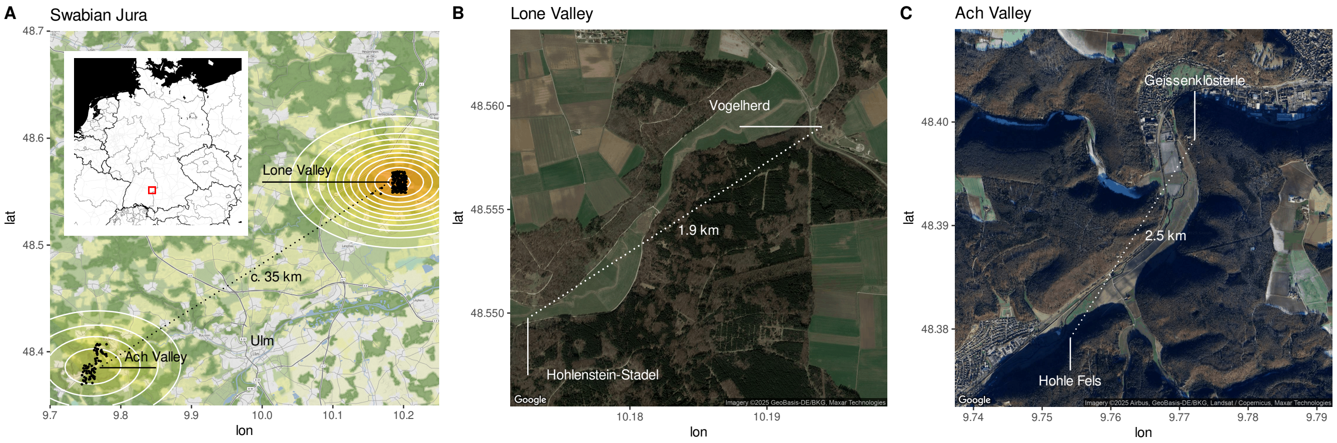

Fig. S1. Cave sites of the Swabian Jura where artefacts with geometric signs were found. **A** Map of the locations of the Ach Valley and Lone Valley in Southwestern Germany. Individual data points (black) indicate objects carrying markings. Density distributions are overplotted. **B** Satellite image of the Lone Valley with locations of the Vogelherd cave and Hohlenstein-Stadel cave. **C** Locations of Geissenklösterle and Hohle Fels cave in the Ach valley.

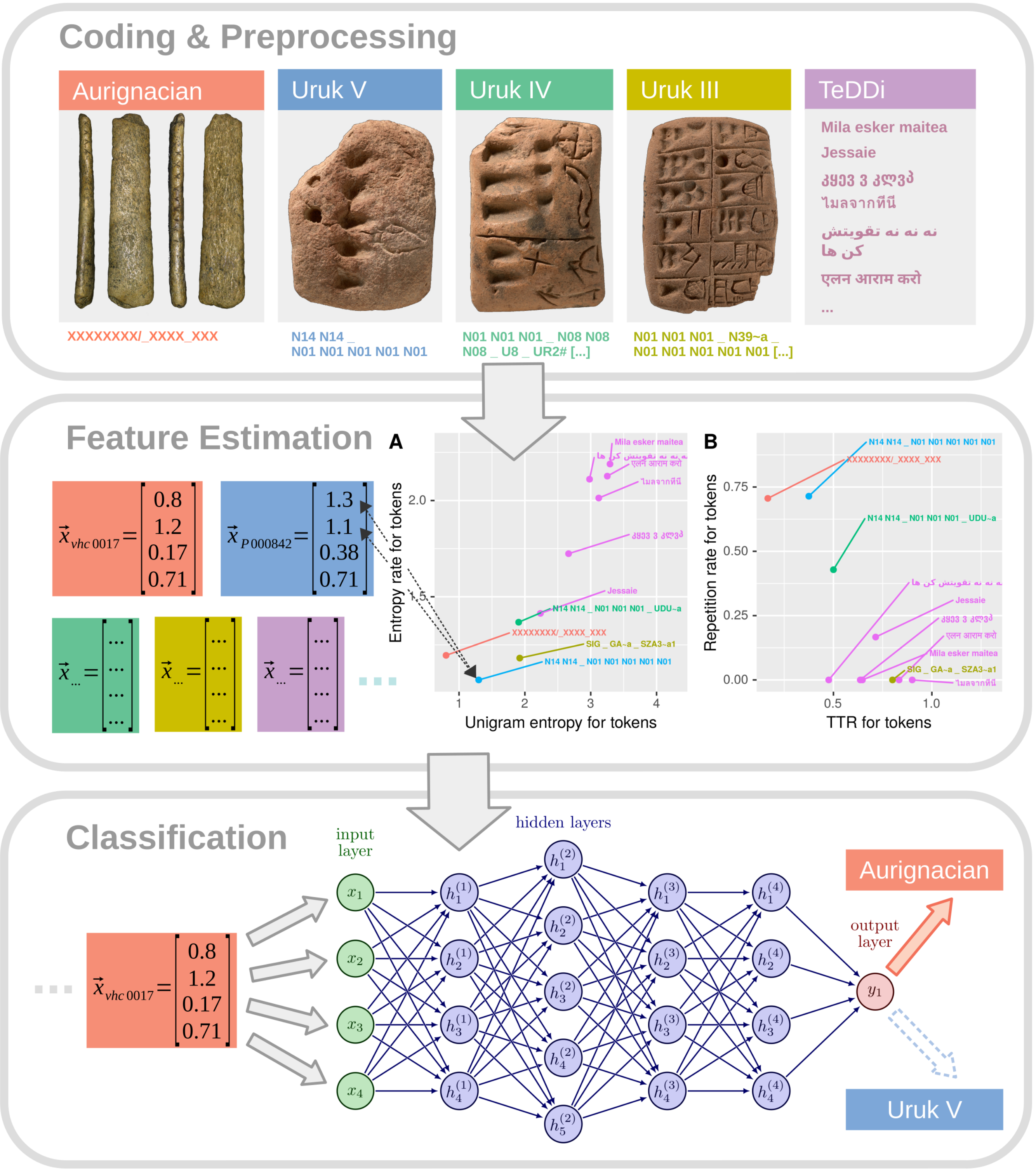

Fig. S2. Workflow of the main analyses. Coding and preprocessing are further described in the Methods Section. Details on feature estimation and classification are also found there. Uruk tablets copyright: CC-BY-SA 4.0, *Staatliche Museen zu Berlin, Vorderasiatisches Museum / Olaf M. Teßmer*.

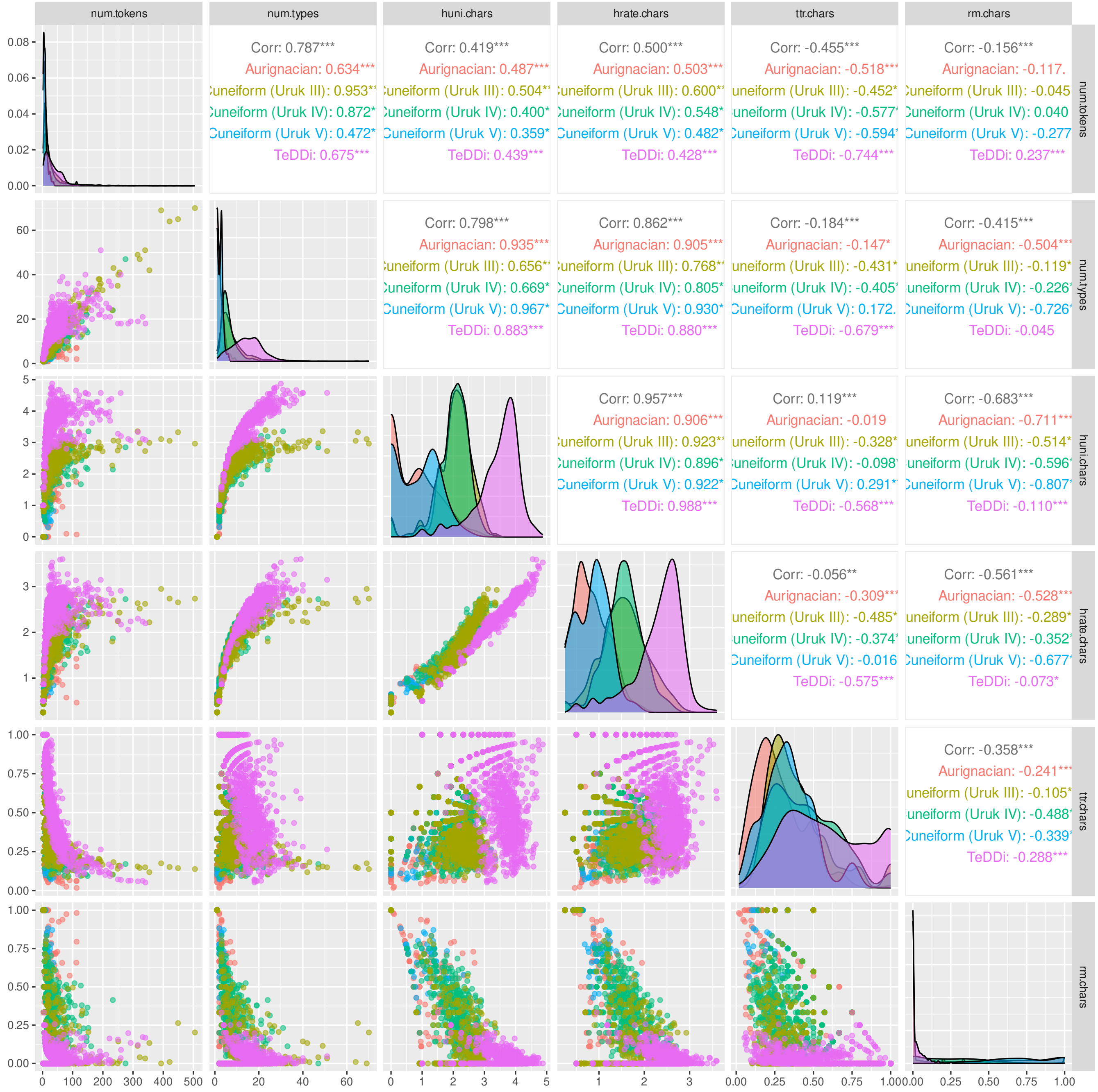

Fig. S3. Correlation plots and density plots. Estimated feature values by subcorpus (i.e. Aurignacian, proto-cuneiform, and TeDDi sample) are given.

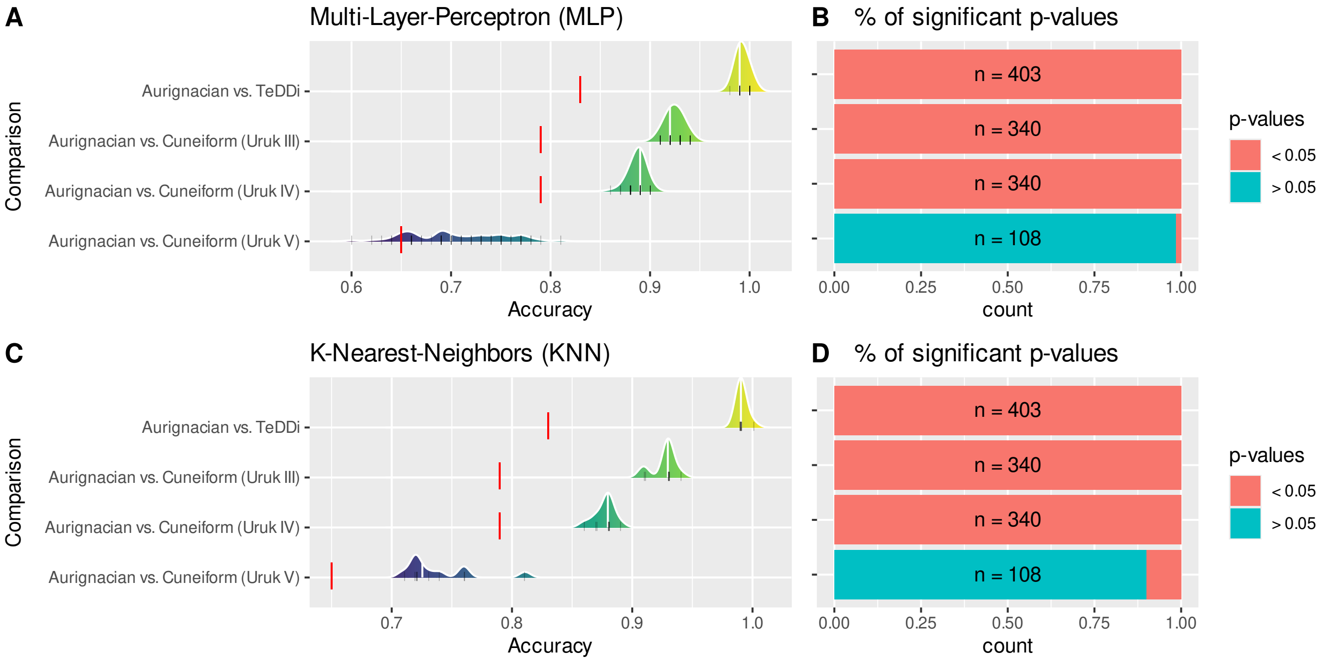

Fig. S4. Classification performance for MLP and KNN methods. **A** Density distributions of accuracy metric for binary classification between Aurignacian sequences versus other sequences (proto-cuneiform and modern writing samples, i.e. TeDDi). Overall up to 100 different MLP architectures are used. Each black tick mark on the x-axes corresponds to the accuracy achieved with a given architecture. Red bars indicate the “no information rate” baseline. **B** Frequencies of p-values below and above the α-level of 0.05 for one-sided binomial tests on accuracies of the MLP classifcations. n is the number of sequences in the test set, i.e. the number of data points for the binomial test. The Bonferroni-correction for multiple testing is applied to the p-values. **C** Binary classification accuracy distributions with the KNN method (for $k = 1$ to $k = 10$). Each tick mark on the x-axes corresponds to the accuracy achieved with KNN given a particular value of k. **D** Frequencies of p-values below and above the α-level of 0.05 for one-sided binomial tests on accuracies of the KNN classifcations. n is the number of sequences in the test set, i.e. the number of data points for the binomial test. The Bonferroni-correction for multiple testing is applied to the p-values.

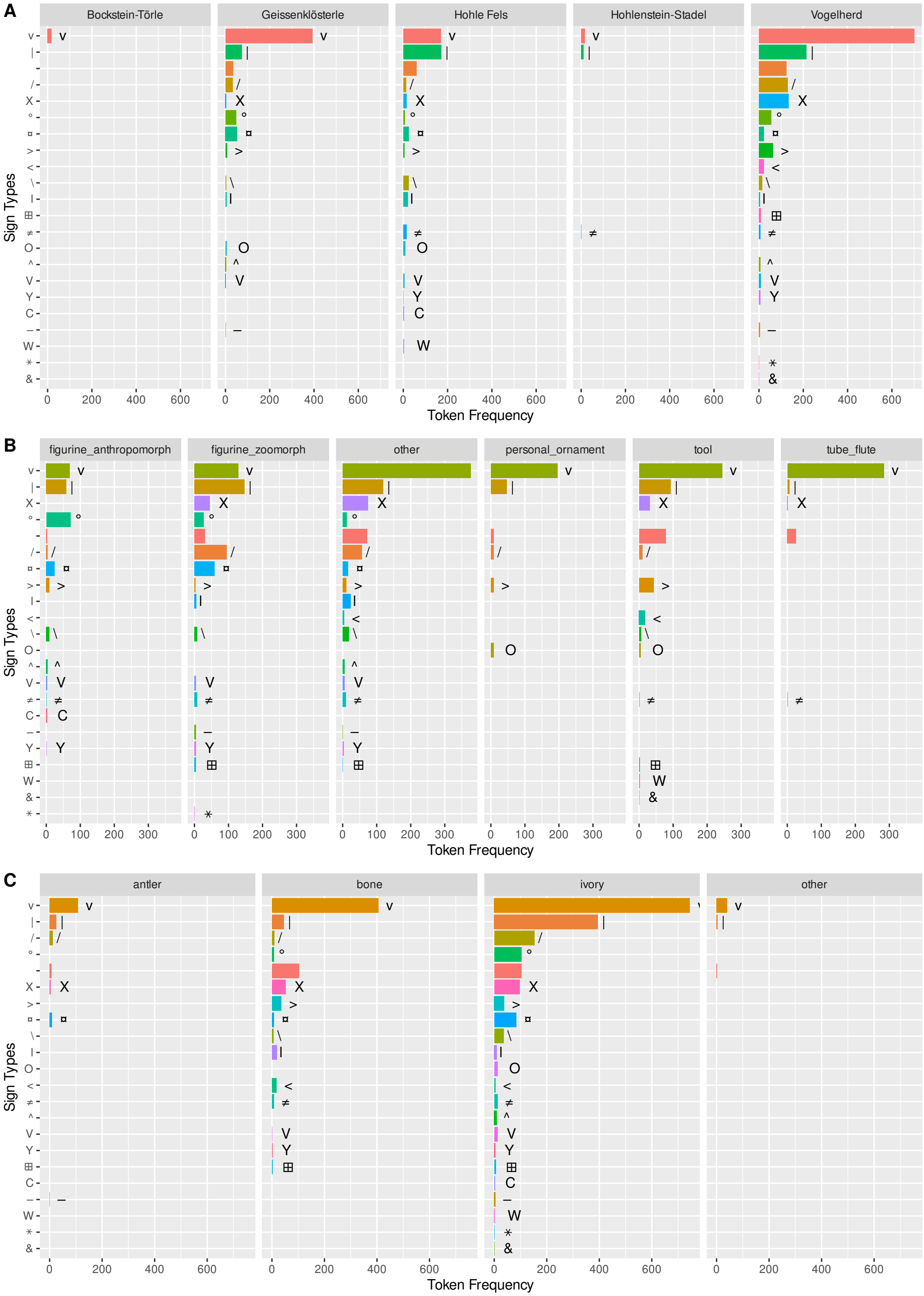

Fig. S5. Distributions of sign type frequencies. **A** Sign type frequencies by cave site. **B** Sign type frequencies by material. **B** Sign type frequencies by object type. Object types are finer-grained in the original data, but are here collapsed into fewer categories.

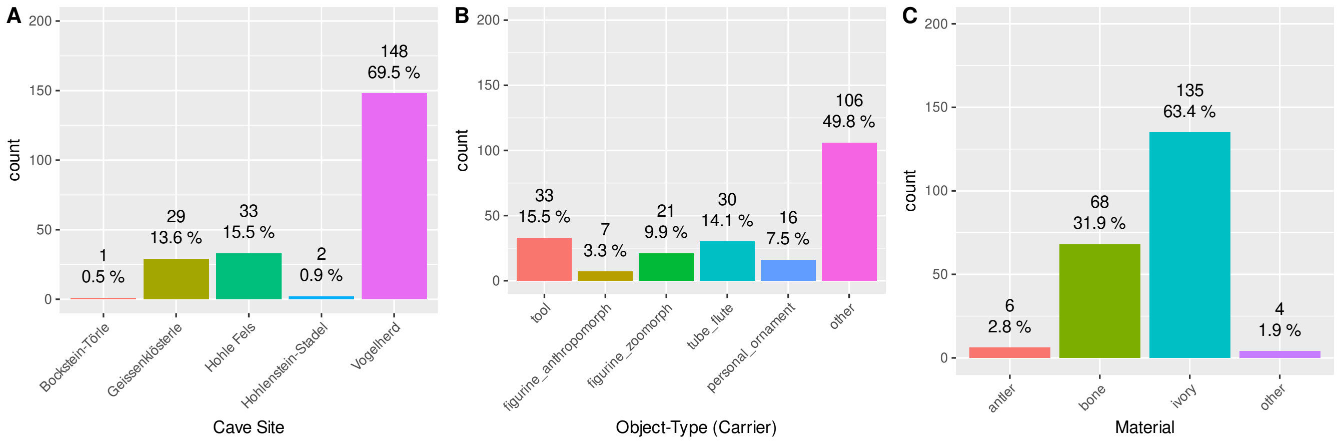

Fig. S6. Barplots with object counts. **A** Counts and percentages of objects per cave site represented in the Swabian Aurignacian sample. **B** Counts and percentages of object types. **C** Counts and percentages of materials.

<table>
<tr><th>ID</th><th>Subcorpus</th><th>Original Sequence</th><th>Preprocessed (Coding clean)</th><th>#Tokens</th><th>Translation</th></tr>
<tr><td>ID426063</td><td>TeDDi (Basque)</td><td>Ez.</td><td>Ez</td><td>2</td><td>No.</td></tr>
<tr><td>ID639415</td><td></td><td>Mila esker, maitea.</td><td>Mila esker maitea</td><td>17</td><td>Thank you very much, dear.</td></tr>
<tr><td>ID5021755</td><td>TeDDi (French)</td><td>J'essaie...</td><td>Jessaie</td><td>7</td><td>I try.</td></tr>
<tr><td>ID5410574</td><td>TeDDi (Georgian)</td><td>კყევ ვ კლუბ?</td><td>კყევ ვ კლუბ</td><td>11</td><td>Do you still have a club?</td></tr>
<tr><td>ID9589824</td><td>TeDDi (Hindi)</td><td>एलन, आराम करो.</td><td>एलन आराम करो</td><td>12</td><td>Alan, relax.</td></tr>
<tr><td>ID10256589</td><td>TeDDi (Indonesian)</td><td>Nenek?</td><td>Nenek</td><td>5</td><td>Grandma?</td></tr>
<tr><td>ID10182648</td><td></td><td>Jangan pernah menyentuhku lagi.</td><td>Jangan pernah menyentuhku lagi</td><td>30</td><td>Don't ever touch me again!</td></tr>
<tr><td>ID10916923</td><td>TeDDi (Japanese)</td><td>{cH00ffff}そうでしょお　母さん?</td><td>そうでしょお　母さん</td><td>10</td><td>What are the differences […]?</td></tr>
<tr><td>ID13125567</td><td>TeDDi (Mandarin)</td><td>不, 不是真的沒有。</td><td>不不是真的沒有</td><td>7</td><td>No, not really.</td></tr>
<tr><td>ID12754029</td><td></td><td>是 我知道 Yeah, I know.</td><td>是 我知道</td><td>5</td><td>Yeah, I know.</td></tr>
<tr><td>ID12754029</td><td>TeDDi (Mandarin, strokes)</td><td>aieeeaetn terduto teetoaie otetaieeeown</td><td>aieeeaetn terduto teetoaie otetaieeeown</td><td>39</td><td>Yeah, I know.</td></tr>
<tr><td>ID14002638</td><td>TeDDi (Persian)</td><td>نه نه نه تقویتش کن، ها؟</td><td>نه نه نه تقویتش کن ها</td><td>21</td><td>No no no, strengthen it, huh?</td></tr>
<tr><td>ID17247600</td><td>TeDDi (Thai)</td><td>60 ไมล์จากที่นี่</td><td>ไมล์จากที่นี่</td><td>10</td><td>60 miles from here</td></tr>
<tr><td>ID19350772</td><td>TeDDi (Vietnamese)</td><td>Để tôi xem vết thương.</td><td>Để tôi xem vết thương</td><td>21</td><td>Let me see the wound.</td></tr>
<tr><td>P000196</td><td>Proto-Cuneiform (Uruk III)</td><td>1. , SIG GA~a SZA3~a1</td><td>SIG _ GA~a _ SZA3~a1</td><td>5</td><td>NA</td></tr>
<tr><td>P000553</td><td></td><td>1. 1(N01) , NAGA~a DU PAP~a [...]</td><td>N01 _ NAGA~a _ DU _ PAP~a</td><td>7</td><td>NA</td></tr>
<tr><td>P005077</td><td></td><td>2.b. , SZUBUR E2~a# SANGA~a#</td><td>NA</td><td>NA</td><td>pigsty accountant</td></tr>
<tr><td>P001084</td><td>Proto-Cuneiform (Uruk IV)</td><td>1. 2(N14) 3(N01) [...] , UDU~a# [...]</td><td>N14 N14 _ N01 N01 N01 _ UDU~a</td><td>8</td><td>20 + 3 sheep (?)</td></tr>
<tr><td>P001282</td><td></td><td>1. 3(N01) 3(N08) , U8 UR2# UMBIN~a […]</td><td>N01 N01 N01 _ N08 N08 N08 _ U8 _ UR2 _ UMBIN~a</td><td>13</td><td>3 + 3 (lamb) ewes, ...;</td></tr>
<tr><td>P001392</td><td></td><td>1.a1. 3(N01) 2(N08) , […] KID~c |SAL.KUR~a| […]</td><td>N01 N01 N01 _ N08 N08 _ […] _ KID~c _ |SAL.KUR~a|</td><td>10</td><td>3 (adult), 2 (child) slaves, […]</td></tr>
<tr><td>P000795</td><td>Proto-Cuneiform (Uruk V)</td><td>1. 3(N01) ,</td><td>N01 N01 N01</td><td>3</td><td>3 (?)</td></tr>
<tr><td>P325364</td><td></td><td>1. 2(N14) 1(N01)? , column 2 2. 7(N01) ,</td><td>N14 N14 _ N01 N01 N01 N01 N01 N01 N01</td><td>10</td><td>20 + 7 (?)</td></tr>
<tr><td>P000852</td><td></td><td>1. 1(N34)# 1(N14)# 4(N01)# , UDU~a X […]</td><td>N34 _ N14 _ N01 N01 N01 N01 _ UDU~a</td><td>10</td><td>60 + 10 + 4 sheep (?)</td></tr>
<tr><td>vhc0096</td><td>Aurignacian (Vogelherd)</td><td>XX! _ vvvvvvvv! _ !vvvv!</td><td>XX _ vvvvvvvv _ vvvv</td><td>16</td><td>NA</td></tr>
<tr><td>vhc0003</td><td></td><td>⊞ _ (||/|/|//|||/||/||| |) _ ⊞ _ || _ ⊞ _ | | | _ |||</td><td>⊞ _ ⊞ _ || _ ⊞ _ | | | _ |||</td><td>18</td><td>NA</td></tr>
<tr><td>hss0001</td><td>Aurignacian (Hohlenstein-St.)</td><td>||||||| _ ≠ _ vvv _ v _ vvvvvvvv _ [!°°°°°°°°°°°°°°°°°°°!]</td><td>||||||| _ ≠ _ vvv _ v _ vvvvvvvv</td><td>24</td><td>NA</td></tr>
<tr><td>hfc0011</td><td>Aurignacian (Hohle Fels)</td><td>|| |||</td><td>|| |||</td><td>6</td><td>NA</td></tr>
<tr><td>gkl0003</td><td>Aurignacian (Geissenklösterle)</td><td>vvvv vvvv</td><td>vvvv vvvv</td><td>9</td><td>NA</td></tr>
</table>

Fig. S7. Examples of original and preprocessed sequences. The character strings affected by preprocessing are marked in red and underlined. Translations for modern languages are provided by https://translate.google.com. The proto-cuneiform translations are taken either directly from the CDLI website, or provided by us with reference to the numeral systems discussed in (12).

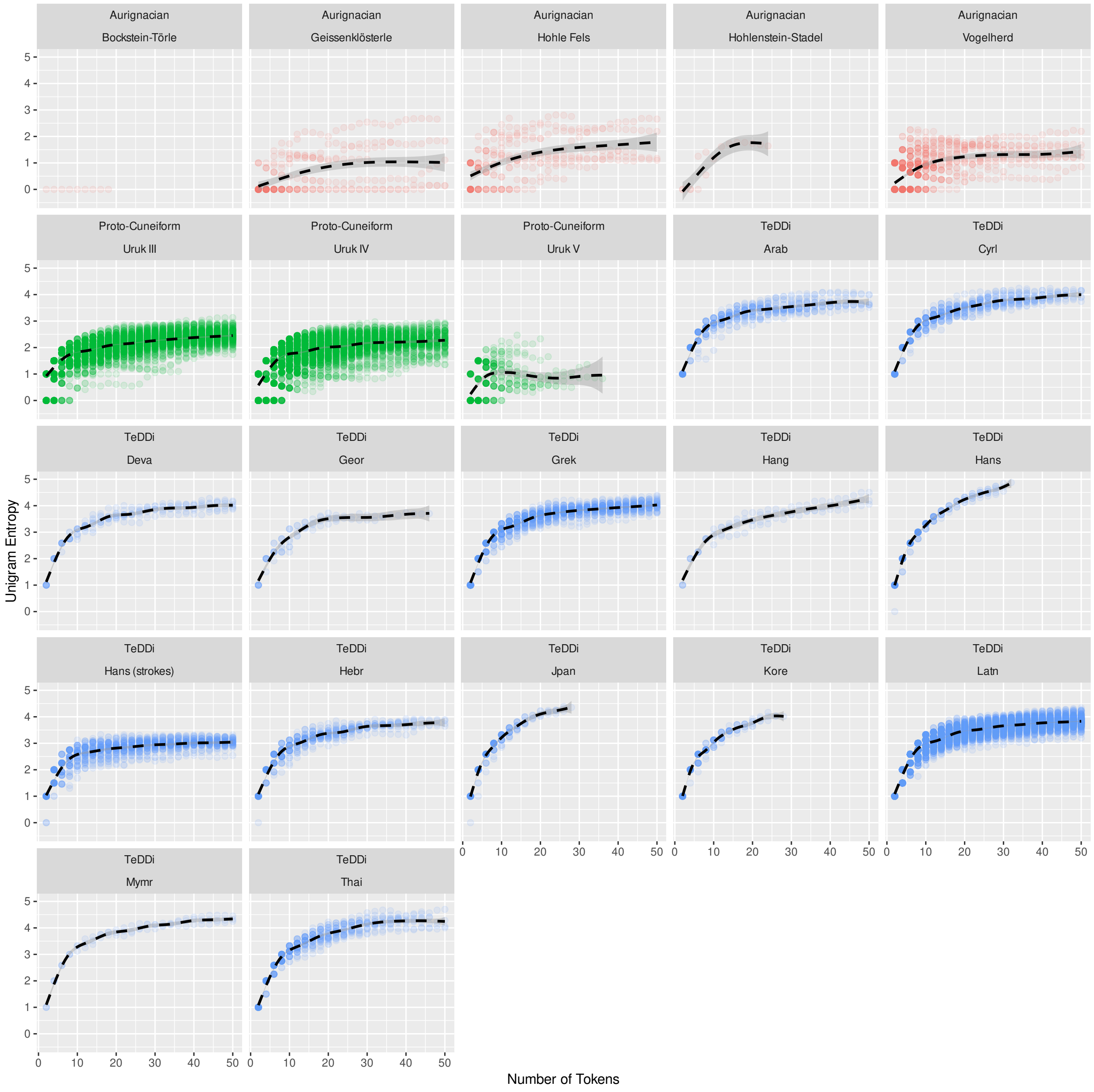

Fig. S8. Stabilization plots for unigram entropies of sequences up to 50 tokens. A local regression smoother is given as dashed black line.

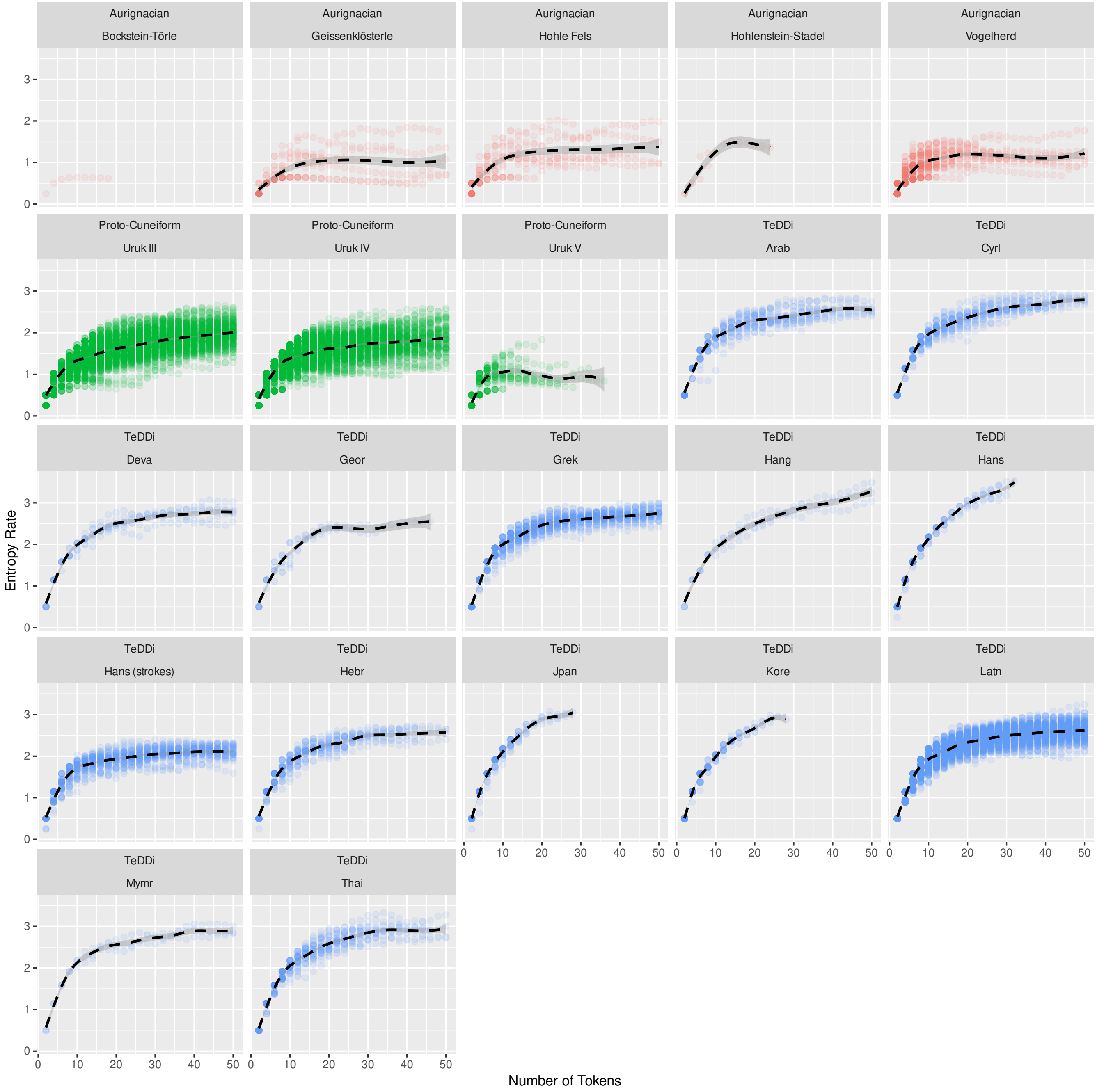

Fig. S9. Stabilization plots for entropy rates of sequences up to 50 tokens. A local regression smoother is given as dashed black line.

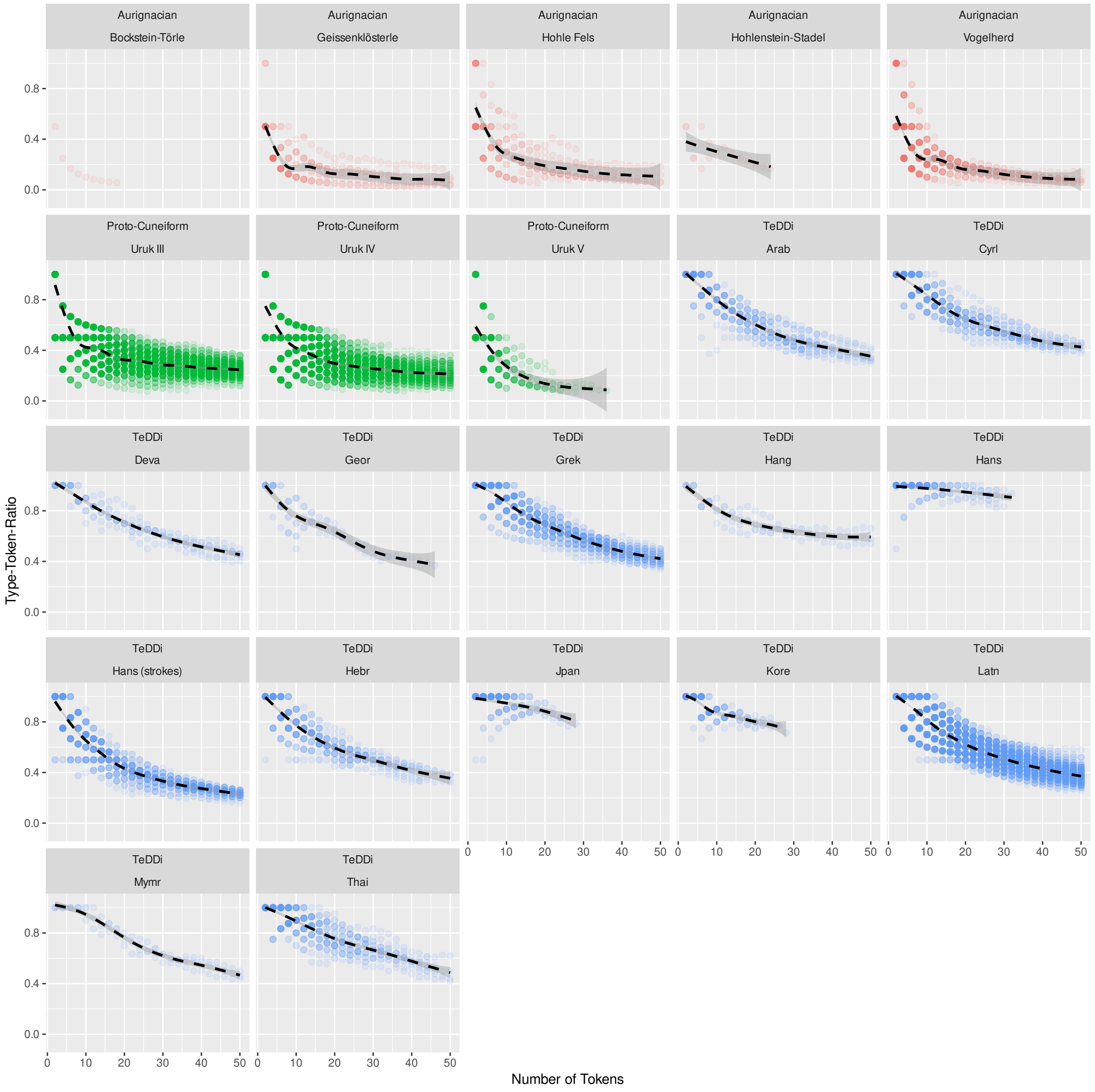

Fig. S10. Stabilization plots for type-token ratios of sequences up to 50 tokens. A local regression smoother is given as dashed black line.

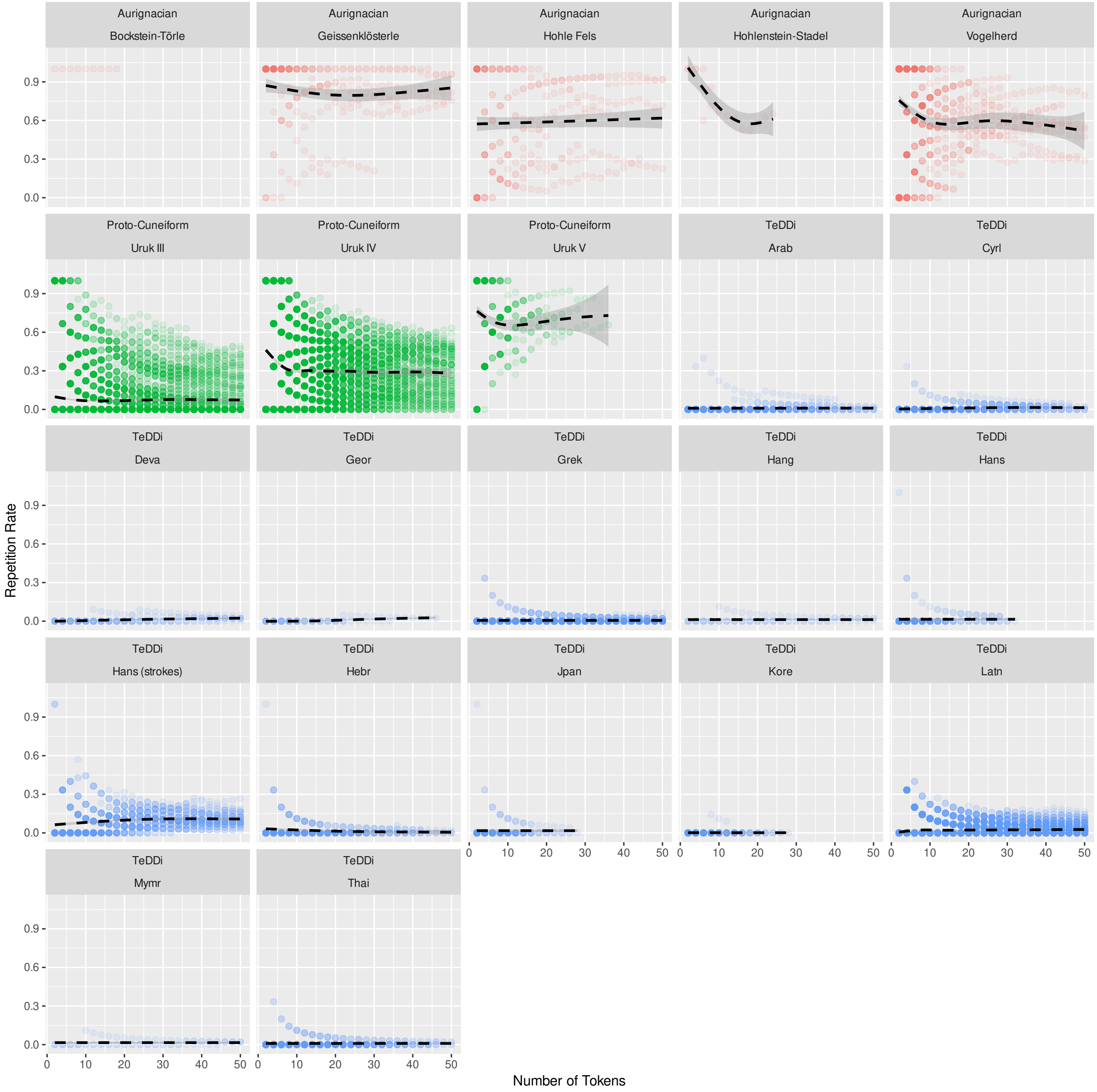

Fig. S11. Stabilization plots for repetition rates of sequences up to 50 tokens. A local regression smoother is given as dashed black line.

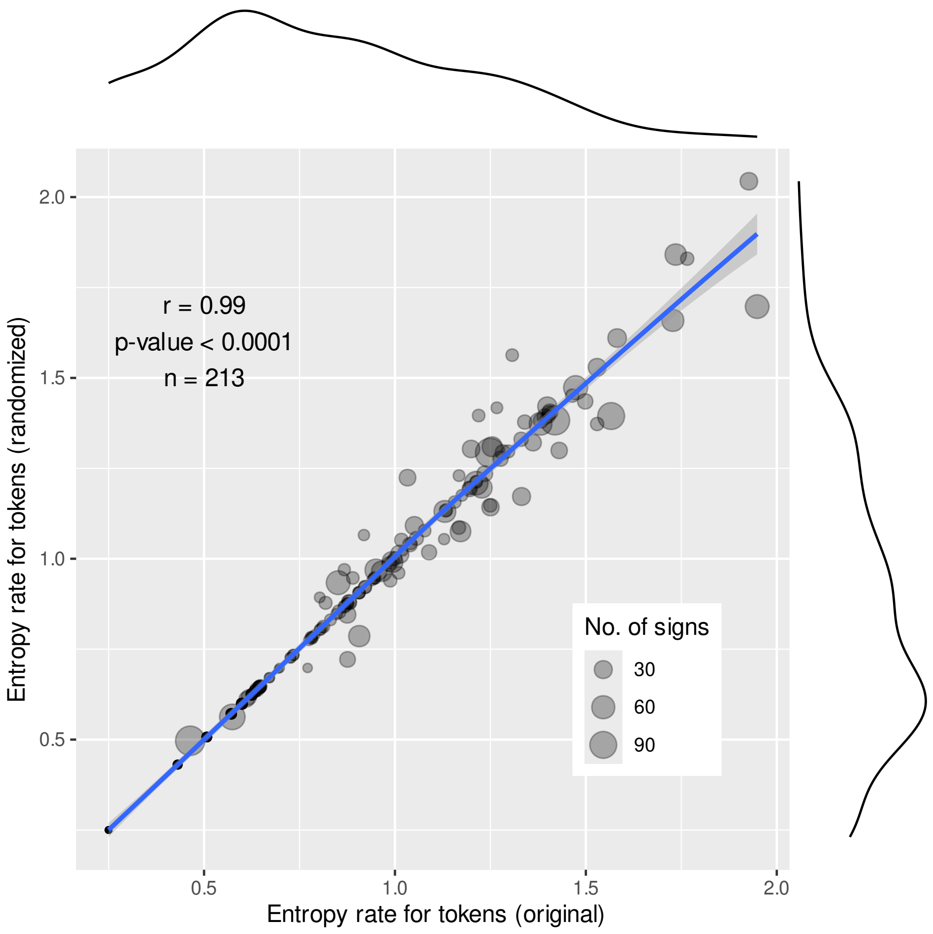

Fig. S12. Plot for entropy rates. Original order of sign strings in sequences of the Swabian Aurignacian vs. randomized strings.

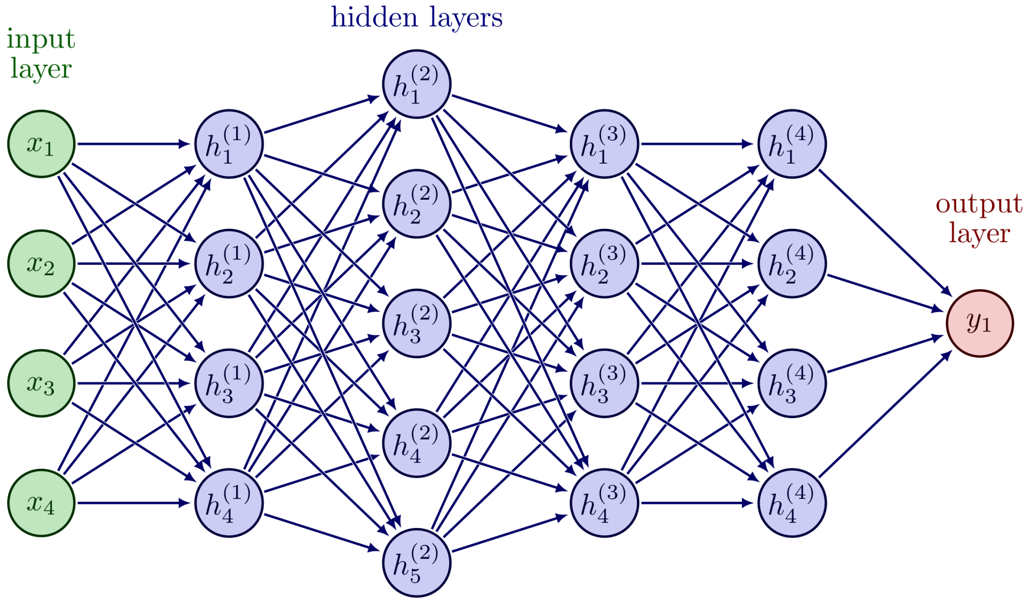

Fig. S13. Most complex MLP architecture. This includes overall 17 hidden units, and 94 parameters (weights and biases). This architecture converged for the binary classification between Aurignacian and UrukV sequences. However, note that this is not the best performing architecture for this task.

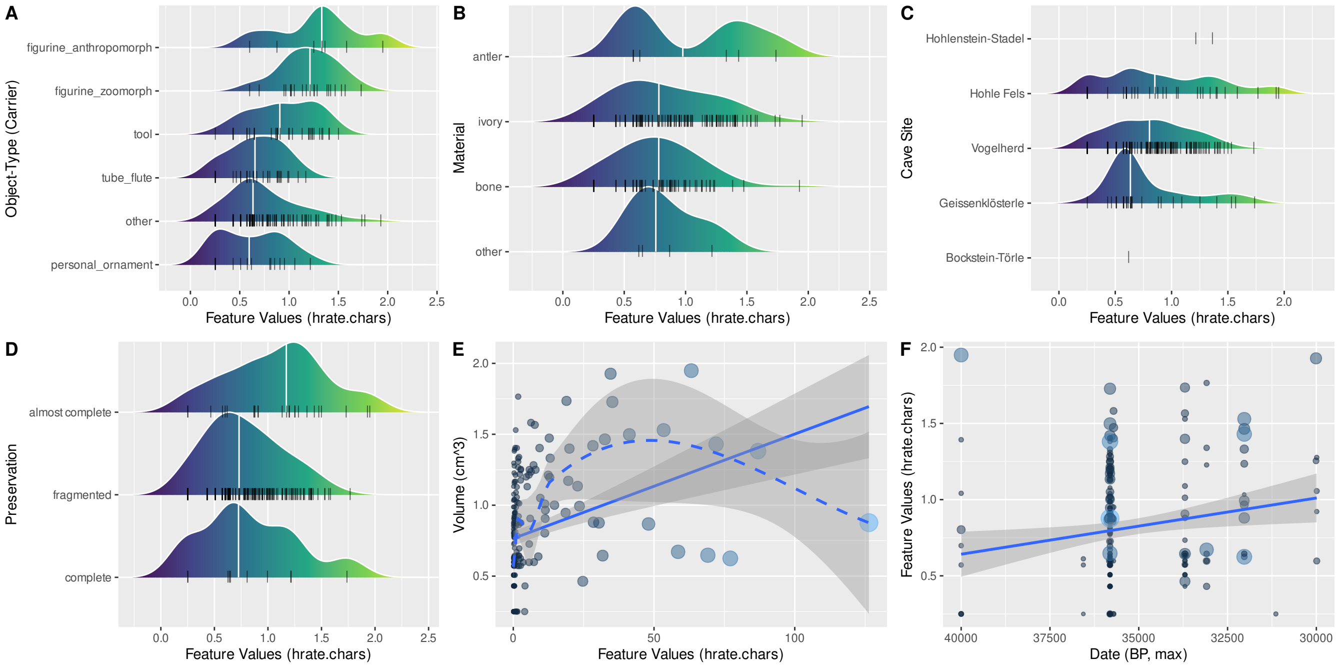

Fig. S14. Visualization of predictor variables for multiple regression model. **A** Statistical feature of entropy rate (x-axis) by object type (factor variable). **B** Entropy rate by material (factor variable). **C** Entropy rate by cave site. **D** Entropy rate by preservation of objects (factor variable). **E** Entropy rate and volume of objects (continuous variable). **F** Entropy rate and age of objects as dates BP (ordinal variable).

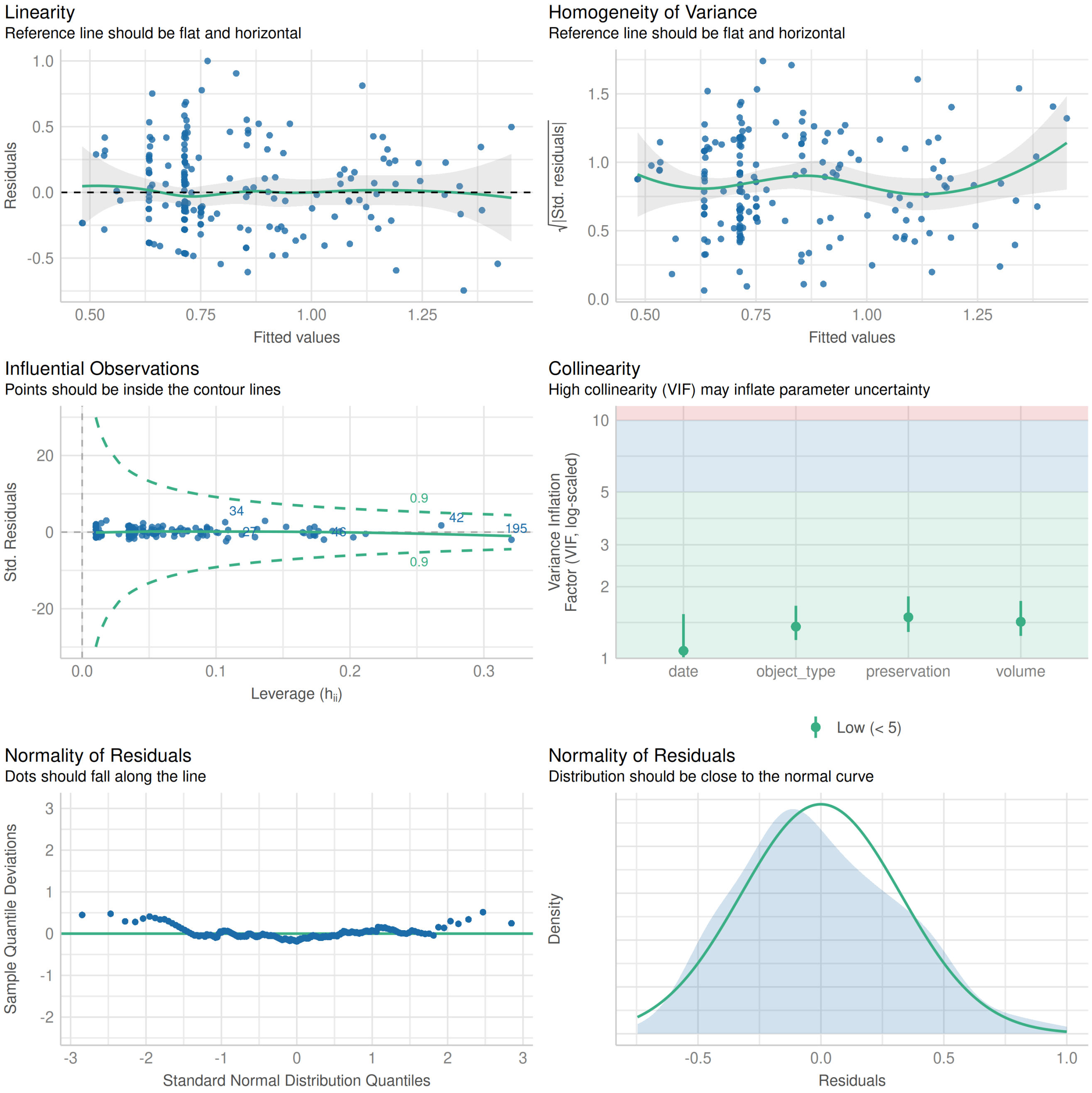

Fig. S15. Panels with visual checks of linear model assumptions.

References

1. E Fix, JL Hodges, Discriminatory analysis - nonparametric discrimination: Small sample performance, (California Univ Berkeley), Technical report (1952).
2. Y Bengio, R Ducharme, P Vincent, A neural probabilistic language model. *Adv. neural information processing systems* **13** (2000).
3. Y LeCun, Y Bengio, G Hinton, Deep learning. *Nature* **521**, 436–444 (2015).
4. A Vaswani, et al., Attention is all you need in *31st Conference on Neural Information Processing Systems (NIPS 2017)*. (Long Beach, CA, USA), (2017).
5. I Goodfellow, Y Bengio, A Courville, *Deep learning.* (MIT press, Cambridge, Massachusetts), (2016).
6. DE Rumelhart, GE Hinton, RJ Williams, Learning representations by back-propagating errors. *Nature* **323**, 533–536 (1986).
7. M Riedmiller, H Braun, A direct adaptive method for faster backpropagation learning: The RPROP algorithm in *IEEE international conference on neural networks.* (IEEE), pp. 586–591 (1993).
8. GE Hinton, S Osindero, YW Teh, A fast learning algorithm for deep belief nets. *Neural computation* **18**, 1527–1554 (2006).
9. C Bentz, The Zipfian challenge: Learning the statistical fingerprint of natural languages in *Proceedings of the 27th Conference on Computational Natural Language Learning (CoNLL)*, eds. J Jiang, D Reitter, S Deng. (Association for Computational Linguistics, Singapore), pp. 27–37 (2023).
10. M Kuhn, Building predictive models in R using the caret package. *J. statistical software* **28**, 1–26 (2008).
11. M Hollander, DA Wolfe, E Chicken, *Nonparametric statistical methods.* (John Wiley & Sons, New Jersey), (2014).
12. RK Englund, Proto-cuneiform account-books and journals in *Creating economic order: Record-keeping, standardization and the development of accounting in the ancient Near East*, eds. M Hudson, C Wunsch. (CDL Press, Bethesda, Maryland, USA), pp. 23–46 (2004).
